# Biomimetic Nano‐delivery of Small‐Molecule Piceatannol Modulates Tumor Stemness and Suppresses Colorectal Cancer Metastasis via Hippo/YAP1/SOX9 Signaling

**DOI:** 10.1002/smll.202407191

**Published:** 2024-11-10

**Authors:** Minfeng Zhou, Huifang Niu, Guoquan Huang, Minquan Zhou, Dandan Cui, Huarong Li, Han Wen, Hongxing Zhang, Fengxia Liang, Rui Chen

**Affiliations:** ^1^ Department of Integrative Chinese and Western Medicine Union Hospital Tongji Medical College Huazhong University of Science and Technology Wuhan 430022 China; ^2^ Jianghan University School of Medicine 8 Triangle Lake Road Wuhan 430056 China; ^3^ Jianghan University Institute of Acupuncture and Moxibustion 8 Triangle Lake Road Wuhan 430056 China; ^4^ Hubei Selenium and Human Health Institute The Central Hospital of Enshi Tujia and Miao Autonomous Prefecture. No.158 Wuyang Avenue Enshi Hubei Province 445000 China; ^5^ Department of Colorectal and Anal Surgery Central Hospital of Enshi Tujia and Miao Autonomous Prefecture. No.158 Wuyang Avenue Enshi Hubei Province 445000 China; ^6^ School of Pharmacy and Nursing Hubei University of Medicine Shiyan 442000 China; ^7^ The Second Affiliated Hospital of Shenyang Medical College 64 West Qishan Road Shengyang 110036 China; ^8^ School of Acupuncture and Bone Injury Hubei University of Traditional Chinese Medicine Wuhan 430065 China

**Keywords:** biomimetic nano‐delivery system, cancer metastasis Hippo/YAP1/SOX9 pathway, colorectal cancer stemness, piceatannol (PTL)

## Abstract

Suppressing tumor metastasis is a crucial strategy for improving survival rates in patients with colorectal cancer (CRC), with cancer stem cells (CSCs) being the primary drivers of metastasis. Current therapeutic approaches targeting CSCs are limited, and their molecular mechanisms remain unclear. To address this challenge, a biomimetic nanoparticle delivery system, CMD‐BHQ3‐PTL/DOX@RBCM is developed, to deliver the stem cell regulator, piceatannol (PTL). This system used carboxymethyl dextran (CMD) and Black Hole Quencher 3 (BHQ3) to encapsulate PTL and the cytotoxic drug doxorubicin (DOX) within a red blood cell membrane (RBCm), enhancing stability and biocompatibility while allowing gradual drug release under hypoxic conditions. The effects of PTL are investigated on CSCs using molecular biology experiments, plasmid construction, and high‐throughput sequencing and elucidated the molecular mechanisms underlying this biomimetic nanoparticle delivery system. The therapeutic efficacy of PTL is validated at the tissue level using subcutaneous and metastatic tumor models in human and murine systems. The results demonstrated that CMD‐BHQ3‐PTL/DOX@RBCM effectively addressed the challenges of specificity and biocompatibility in vivo, significantly inhibiting CSC‐related tumor metastasis. This inhibitory effect is closely associated with the Hippo/YAP1/SOX9 pathway. This study highlights the effectiveness of the pH‐responsive biomimetic nanoparticle system CMD‐BHQ3‐PTL/DOX@RBCm in delivering PTL to tumor sites, with SOX9 and its upstream Hippo/YAP1 pathway playing a critical role in the underlying mechanism.

## Graphical abstract

This study demonstrates that CMD‐BHQ3‐PTL nanoparticles selectively target and inhibit colorectal cancer stemness by modulating the tumor microenvironment, enhancing therapeutic efficacy. Results show that encapsulated piceatannol synergizes with PTL, effectively suppressing cancer cell stemness markers and reducing metastatic potential, underscoring the promise of this nanoparticle delivery system for targeted cancer therapy.

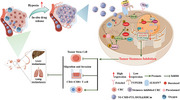

## 1 Introduction

Colorectal cancer (CRC) is the third most common malignancy globally. Approximately 21%–26% of patients progress to metastatic disease, which is the primary cause of death.^[^ ^1^ ^]^ Patients with early‐stage CRC who undergo complete surgical resection achieve five‐year survival rates ranging from 30% to 57%.^[^ ^2^ ^]^ However, the five‐year survival rate is <5% for patients with metastatic CRC (mCRC).^[^ ^3^ ^]^ Tumor stemness is the primary driving force behind metastasis.^[^ ^4^ ^]^ Therefore, it is crucial to prioritize the development of treatments that effectively suppress tumor stemness in CRC therapy.^[^ ^5^ ^]^ Nevertheless, current biotherapeutics have been marred by low tissue compatibility and inadequate specificity.^[^ ^6^ ^]^ Crafting innovative interventions that distinctly target and inhibit tumor stemness and overcome the limitations inherent in existing treatments holds tremendous promise.

In mCRC research, gaining a profound understanding of the regulatory mechanisms of tumor stemness is of paramount importance.^[^ ^7^ ^]^ This regulation involves the collaborative actions of intracellular molecular mechanisms and external signaling pathways, including Wnt/β‐catenin,^[^ ^8^ ^]^ Notch,^[^ ^9^, ^10^, ^11^ ^]^ and Hedgehog pathways,^[^ ^12^ ^]^ along with associated epigenetic modifications,^[^ ^13^ ^]^ activation or inhibition of transcription factors,^[^ ^7^, ^8^, ^14^ ^]^ and cell cycle regulation.^[^ ^15^, ^16^ ^]^ These mechanisms play a crucial role in maintaining tumor stemness and may lead to abnormal activation,^[^ ^17^ ^]^ drive tumor growth and dissemination,^[^ ^18^ ^]^ and directly impact the survival and proliferation of tumor cells.^[^ ^19^, ^20^ ^]^ Phenotypic heterogeneity of tumor stemness is a unique characteristic,^[^ ^21^ ^]^ manifested by the existence of multiple subgroups within tumors, each possessing different functions and characteristics. This heterogeneity complicates tumor response to treatment, as different subgroups may exhibit varied responses.^[^ ^22^ ^]^ The impact of therapeutic interventions on tumor stemness is another extensively studied aspect of mCRC research.^[^ ^23^ ^]^ Different treatment strategies may exert varying degrees of influence on tumor stemness, including inhibition of proliferation and reduction of drug resistance.^[^ ^24^ ^]^ Approaches such as chemotherapy, targeted therapy, and immunotherapy can achieve therapeutic effects by disrupting the molecular mechanisms of tumor stemness.^[^ ^15^, ^24^ ^]^ However, the current focus is to explore novel therapeutic methods targeting tumor stemness driven by numerous side effects, drug resistance, poor drug targeting specificity, and low tumor tissue specificity.

The monomeric compound piceatannol (PTL), extracted from traditional Chinese medicine, has recently garnered significant attention, particularly concerning CRC and inhibiting tumor stemness.^[^ ^25^, ^26^ ^]^ PTL, a widely used anticancer agent, has demonstrated efficacy in treating various malignancies, such as gastric,^[^ ^27^ ^]^ Pancreas,^[^ ^28^ ^]^ non‐small cell lung,^[^ ^29^ ^]^ and Rolorectal Cancers.^[^ ^26^, ^30^ ^]^ Its mechanism of action is multifaceted, involving augmentation of microtubule stability, induction of cell cycle arrest,^[^ ^31^ ^]^ initiation of apoptosis,^[^ ^32^ ^]^ enhancement of immune responses,^[^ ^33^ ^]^ inhibition of angiogenesis,^[^ ^34^ ^]^ and disruption of intercellular signaling pathways.^[^ ^27^, ^28^, ^31^, ^32^, ^34^ ^]^ TL, a natural product, is deemed to possess remarkable anticancer potential, particularly in suppressing tumor stemness. Nevertheless, several research challenges persist.^[^ ^25^, ^35^ ^]^ First, a deeper understanding of the molecular mechanisms underlying PTL inhibition of CRC and regulation of tumor stemness is warranted, necessitating detailed molecular‐level investigations to elucidate its precise pathways.^[^ ^25^ ^]^ Second, despite exhibiting relatively good biological activity, issues related to PTL bioavailability and pharmacokinetics require further exploration.^[^ ^26^ ^]^ Additionally, comprehension of PTL's in vivo distribution and metabolic processes remains limited but holds significant implications for its development as a potential anticancer drug.^[^ ^35^ ^]^ To address these challenges, we advocate a comprehensive exploration of PTL molecular‐level mechanisms while concurrently tackling issues surrounding its bioavailability and pharmacokinetics. A thorough understanding of PTL's in vivo metabolism and distribution may provide a more robust foundation for its potential development as an anticancer drug.

To address these challenges, we introduced an innovative pH‐responsive biomimetic nano‐delivery system to enhance the local delivery efficacy of the monomeric drug PTL. This system, CMD‐BHQ3‐PTL/DOX@RBCM, is based on carboxymethyl dextran (CMD) and Black Hole Quencher 3 (BHQ3). It further encapsulates doxorubicin (DOX) and PTL through red blood cell membrane (RBCM) wrapping to improve biological tissue compatibility.^[^ ^36^ ^]^ Initially, our study demonstrated the interventional effect of PTL on tumor stemness through immunoblotting to overcome current treatment deficiencies, especially in high oxygen demand and low pH environments in CRC.^[^ ^37^ ^]^ We investigated the molecular mechanisms of the biomimetic nano‐delivery system at the cellular level concerning tumor stemness‐related metastasis by characterizing nanomaterials and employing molecular biology experiments, plasmid construction, and high‐throughput sequencing. Subsequently, our research was extended to a more complex tissue level by constructing subcutaneous tumor and metastatic models in humans and mice. This comprehensive approach allowed us to elucidate the molecular mechanisms of biomimetic nano‐delivery system in greater depth.

## 2 Results

### 2.1 Stemness Suppression Emerges as the Predominant Mechanism in PTL‐Inhibited CRC

**Figure 1:**
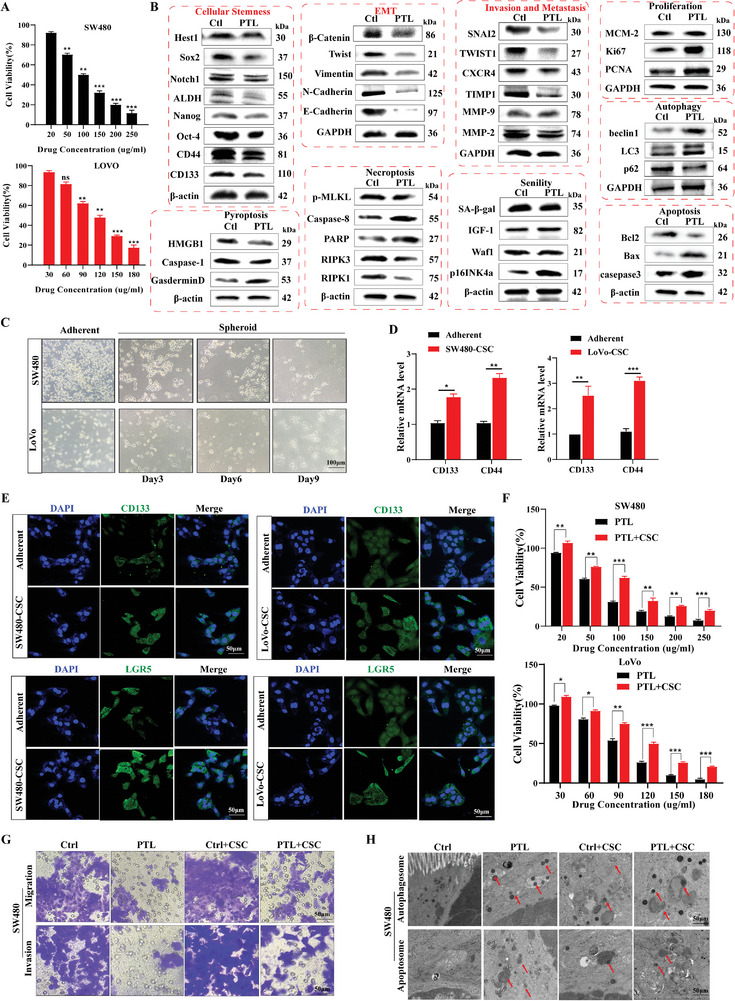
Stemness suppression is the predominant mechanism in PTL‐inhibited CRC. A) Evaluation of SW480 and LoVo cell viability after 48 h of PTL treatment. B) PTL suppressed cellular stemness, prevented EMT, and impeded tumor invasion and metastasis. Nonetheless, PTL did not significantly regulate necrosis, pyroptosis, autophagy, senescence, proliferation, and apoptosis. C) Observation of pellet formation of CRC cells cultured in tumor stem cell medium using optical microscopy (scale bar: 100 µm; *n* = 3). D) The expression of stemness‐related factors, CD133 and CD44, in PTL‐treated SW480 and LoVo cells, was assessed using qPCR. E) Immunofluorescence was employed to validate the expression levels of stemness‐related factors, CD133 and LGR5, in SW480 and LoVo cells treated with PTL (scale bar: 50 µm; *n* = 3). E) Quantifying cell viability in SW480 and LoVo cells induced by the stem cell culture medium using qPCR. F) Evaluation of the viability of SW480 cells induced by stem cell culture medium after 48 h of PTL treatment. G) The effect of PTL on SW480 cells induced by the stem cell culture medium was confirmed using cell invasion and migration assays (scale bar: 50 µm; *n* = 3). H) TEM was used to verify the influence of PTL on apoptosis induction in SW480 cells cultured in the stem cell culture medium (scale bar: 50 µm; *n* = 3). Statistical results are presented as the mean ± SD using a student's t‐test or one‐way ANOVA. * *P* < 0.05; **P < 0.01; ***P < 0.001.

The cytotoxicity of PTL against SW480 and LoVo cells was assessed using an MTT assay (**Figure** **1**A). The results revealed that PTL effectively inhibited the viability of both cell types in a dose‐dependent manner. The half maximal inhibitory concentration (IC50) values of PTL in SW480 and LoVo cells were 97.44 ± 1.18 and 107.33 ± 0.97 µg mL^−1^, respectively (P < 0.05). These findings indicated that PTL exhibits comparable cytotoxic effects in both cell lines. Subsequent mechanistic investigations unveiled that PTL can inhibit cellular stemness and epithelial‐mesenchymal transition (EMT) at tumor invasion and metastasis onset. Cellular stemness underpins several other phenotypes (Figure 1B; Figure S2A–C, Supporting Information). According to previous research,^[^ ^26^, ^30^ ^]^ PTL notably induces autophagy and apoptosis in LoVo cells. However, its induction effect on senescence, proliferation, pyroptosis, and necrotic apoptosis remains inconspicuous(Figure 1B). Subsequently, we initiated the differentiation of SW480 cells into stem‐like SW480 cells (SW480‐CSCs) on the 9th day and LoVo cells into stem‐like LoVo cells (LoVo‐CSCs) on the 6th day using a tumor stem cell culture medium (Figure 1C). The expression levels of CD133, LGR5, and CD44, representing stemness, were evaluated using quantitative polymerase chain (qPCR; Figure 1D) and immunofluorescence (Figure 1E). The outcomes confirmed the successful induction of stemness in SW480‐CSCs and LoVo‐CSCs.

Furthermore, we employed PTL to intervene with tumor cells, including those with stem‐like properties. The MTT results (Figure 1F) demonstrated that, at equimolar doses, stem‐like tumor cells were significantly less susceptible to PTL‐induced cell death than free PTL (*P* < 0.05), suggesting the potential role of tumor cell stemness in PTL cytotoxicity. The evaluation of invasion and migration of tumor cells morphologically confirmed that PTL inhibited tumor cell migration and invasion by intervening with cell stemness (Figure 1G). The formation of autophagosomes and apoptotic bodies represents major morphological changes during autophagy‐related apoptosis in cells (Figure 1G). Following PTL intervention, cells displayed autophagosomes, characterized by double‐membrane structures, varying sizes, encapsulated contents, and fusion or release with lysosomes. Additionally, irregular apoptotic bodies with vesicular structures and abundant cellular fragments were observed within cells. These changes were less prominent in the stem‐like tumor cells (*P* < 0.05). These results suggest stemness inhibition is a key mechanism underlying PTL‐induced effects in CRC.

PTL uniquely regulates tumor stem cells, selectively targets tumor microenvironments, and demonstrates compatibility with the CMD‐BHQ3 delivery system.^[^ ^37^ ^]^ By inhibiting tumor stem cell activity, PTL disrupts cancer cell hierarchies, enhancing therapeutic efficacy. Additionally, PTL modifies the interactions between C (CAFs) and tumor cells, diminishing CAFs' tumor‐promoting effects.^[^ ^35^ ^]^ Its compatibility with the CMD‐BHQ3 system further enhances bioavailability and controlled release, optimizing tumor‐targeted delivery and minimizing adverse effects.^[^ ^37^, ^38^ ^]^ Thus, PTL stands out as a promising candidate for colorectal cancer therapy, combining stemness inhibition with innovative delivery strategies.

### 2.2 Preparation and Characterization of Nanoparticles

Our recent emphasis is to explore a drug delivery system based on nanocarriers to augment PTL's therapeutic potential in targeting cellular stemness. Hypoxia is a well‐established adversary in cancer treatment that fosters chemoresistance, radioresistance, angiogenesis, invasiveness, and metastasis.^[^ ^39^, ^40^ ^]^ Therefore, our research focused on HR‐NP synthesis.

**Figure 2:**
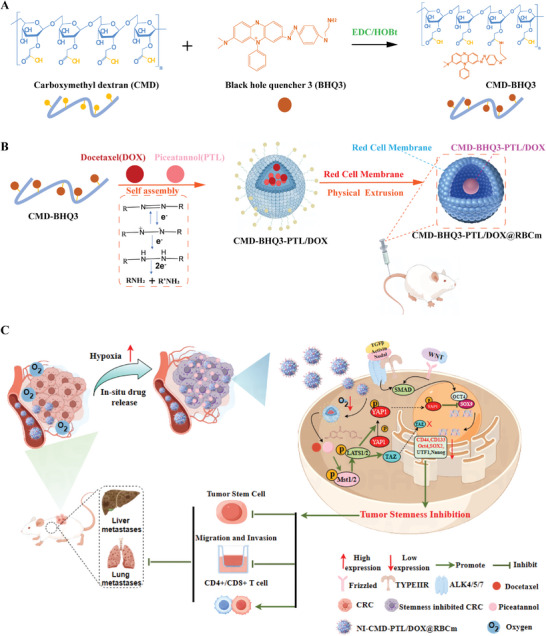
Synthesis of CMD‐BHQ3‐PTL/DOX@RBCM and mechanism of cancer treatment by targeting cancer stemness. A) Synthesis of nanoparticles. B) Drug loading of the nanoparticles. C) Schematic representation of the mode of action of the nanomedicine.

Scheme 1 and Figure S1 (Supporting Information) depict the synthesis of the most fundamentally self‐assembled HR‐CMD‐NPs. We refined the synthesis approach to produce CMD‐BHQ3 (**Figure** **2**A) based on HR‐CMD‐NPs. Subsequently, we encapsulated PTL and DOX within CMD‐BHQ3, forming HR‐NPs (CMD‐BHQ3‐PTL and CMD‐BHQ3‐PTL/DOX; Figure 2B,C).

**Figure 3:**
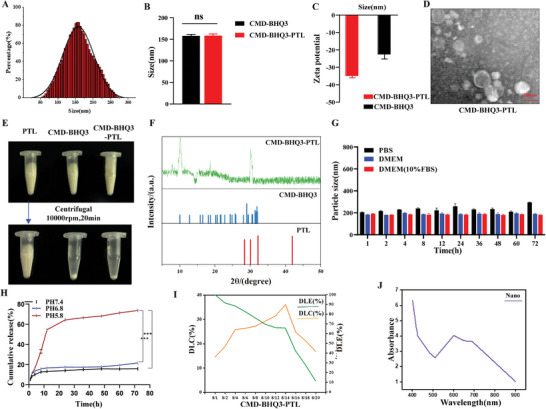
Preparation and characterization of CMD‐BHQ3‐PTL. A) Size distribution of CMD‐BHQ3‐PTL, as measured by DLS (*n* = 3). B) Average particle sizes of CMD‐BHQ3, and CMD‐BHQ3‐PTL(*n* = 3). C) Zeta potentials of CMD‐BHQ3, and CMD‐BHQ3‐PTL. D) TEM images of CMD‐BHQ3‐PTL(*n* = 3, scale bar: 200 nm). E) Centrifugal results for PTL, CMD‐BHQ3, and CMD‐BHQ3‐PTL aqueous solutions. F) PXRD analysis of the diffraction peaks of PTL, CMD‐BHQ3, and CMD‐BHQ3‐PTL. G) Stability analysis of CMD‐BHQ3‐PTL in the presence of proteins and salt ions(*n* = 3). H) In vitro PTL release from CMD‐BHQ3‐PTL in PBS (pH 5.8, 6.5, and 7.4) (*n* = 3). I) DLE and DLC results of nanoparticle CMD‐BHQ3‐PTL under different PTL/CMD‐BHQ3 mass ratios(*n* = 3). J) The UV–vis absorption spectrum of CMD‐BHQ3‐PTL shows distinct peaks at 594.15 ± 2.35 nm, indicating successful encapsulation of piceatannol (PTL). Data were obtained under controlled conditions and are presented as the mean of three independent measurements (*n* = 3). Statistical results are presented as the mean ± SD using a student's t‐test or one‐way ANOVA.

**Figure** **3**A,B and Figure S3A,B (Supporting Information) present the results of DLS measurements. The particle sizes of CMD‐BHQ3 and CMD‐BHQ3‐PTL were 156.18 ± 0.34 and 155.43 ± 0.48 nm, respectively. The zeta potentials for CMD‐BHQ3 and CMD‐BHQ3‐PTL were −21.51 ± 0.33 and −33.59 ± 0.74 mV, respectively (Figure 3C). TEM validated these findings, demonstrating consistent sizes of ≈156 and 155 nm for CMD‐BHQ3 and CMD‐BHQ3‐PTL, respectively (Figure S3B,D, Supporting Information). Following water dissolution and subsequent centrifugation of PTL, CMD‐BHQ3, and CMD‐BHQ3‐PTL, the outcomes confirmed the effective incorporation of PTL into CMD‐BHQ3, resulting in the formation of CMD‐BHQ3‐PTL particles (Figure 3E).

In vitro stability tests were conducted in two different culture media, PBS and FBS. These findings suggest that CMD‐BHQ3‐PTL demonstrates enhanced serum stability compared to PTL and the feasibility of prolonged storage in PBS (Figure S3C, Supporting Information). Various formulations of CMD‐BHQ3‐PTL were obtained by altering the mass ratio of PTL to CMD‐BHQ3, resulting in diverse drug loading efficiencies (DLE) and DLC (Table S1, Supporting Information). Given the outcomes of DLE and DLC, we opted for a PTL/CMD‐BHQ3 mass ratio of 8/8 to synthesize CMD‐BHQ3‐PTL (with a DLE of 76.1% and DLC of 27.2%) for subsequent surface modification and characterization. After synthesizing the nanoparticles at various feed ratios, their particle sizes (Figure 3I; Figure S3D, Supporting Information) and zeta potentials (Figure S3E, Supporting Information) were assessed. The findings revealed that as the PTL/CMD‐BHQ3 feed ratio increased, the zeta potential of the CMD‐BHQ3‐PTL initially decreased and then increased. Given that nanoparticles ranging in size from 20 to 200 nm are more favorable to achieve stable in vivo circulation, and a lower zeta potential contributes to prolonged circulation time, we opted for conditions with a feed ratio of 8/8 to fabricate CMD‐BHQ3‐PTL, which was subsequently employed for further characterization and experimentation.

Moreover, PXRD analysis spectra revealed that CMD‐BHQ3‐PTL displayed broader diffraction peaks than CMD‐BHQ3 and PTL, with distinctive peaks observed at 10, 18, and 30. These findings indicated that incorporating PTL did not significantly impact the CMD‐BHQ3 structure (Figure 3F). Furthermore, we assessed the stability of CMD‐BHQ3‐PTL in the presence of proteins and salt ions (Figure 3G). These findings indicated that CMD‐BHQ3‐PTL maintained negligible alterations in particle size even after 72 h of incubation in PBS, DMSO, or a DMSO solution containing 10% FBS. This underscores its superior stability in blood circulation and effective accumulation in tumor tissues. Moreover, investigations of the in vitro drug release characteristics indicated that the slight acidity present at the tumor site could accelerate the release of PTL from CMD‐BHQ3‐PTL, highlighting the potential efficacy of this nanocarrier system in tumor therapy (Figure 3H). Moreover, we analyzed the optical absorption features of the nanomaterials using UV–vis spectroscopy, conclusively confirming the effective encapsulation of PTL within the CMD‐BHQ3‐PTL (Figure 3J).

### 2.3 Cellular Uptake of CMD‐BHQ3‐PTL for Modulating Stemness in CRC Cells

**Figure 4:**
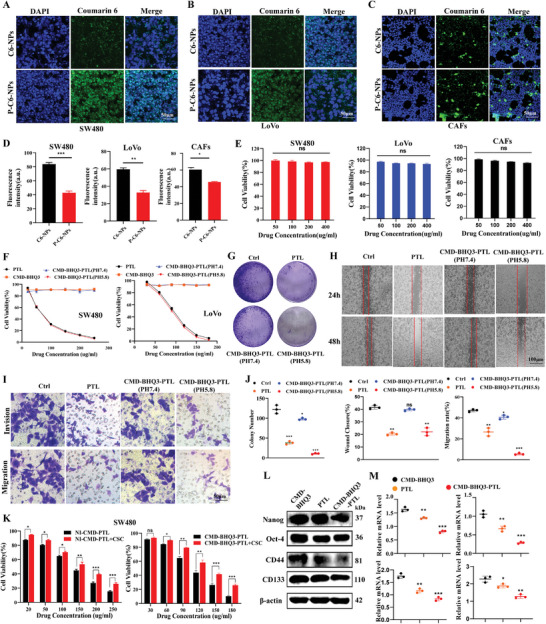
Cellular uptake of CMD‐BHQ3‐PTL for modulating stemness in CRC cells. Uptake of C‐NP and P‐C6‐NP by A) SW480 cells, B) LoVo cells, and C) CAFs. D) Quantitative results of the fluorescence intensity in tumor cells. E) Safety assessment was conducted on the normal colon epithelial cell line NCM460 and CRC cell line SW480/LoVo(*n* = 3). F) MTT assay of drug intervention in SW480 and LoVo Cells(*n* = 3). G) Colony formation assay results(*n* = 3). H) CRC cell scratch assay results. I) CRC cell migration and invasion assay results(*n* = 3). J) Statistical diagram of the colony formation, cell invasion, and cell scratch experiments(*n* = 3). K) MTT assay results for CRC cells after treatment with nanoparticles following cancer stem cell (CSC) medium intervention(*n* = 3). L) Western blotting (WB) to detect cell stemness‐related proteins after nanoparticle and PTL intervention(*n* = 3). M) Reverse transcription (RT)‐qPCR to detect stemness‐related gene expression(*n* = 3). Statistical results are presented as the mean ± SD using a student's t‐test or one‐way ANOVA. *P < 0.05; **P < 0.01; ***P < 0.001.

C6 was employed as a fluorescent label to track the intracellular uptake of the nanoparticles. Following a short 6‐h co‐incubation, both CRC cell lines (SW480/LoVo) and cancer‐associated fibroblasts (CAFs) displayed green fluorescence indicative of the absorption of C6‐NPs (CMD‐BHQ3) or P‐C6‐NPs (CMD‐BHQ3‐PTL). This observation suggests effective infiltration of nanoparticles into the cells (**Figure** **4**A–C). Figure 4D depicts a semi‐quantitative analysis. Safety assessments were performed on the NCM460 normal colorectal epithelial cell line and CRC cell lines SW480/LoVo using elevated concentrations of blank nanoparticles (CMD‐BHQ3). No discernible toxicity was observed in the three cell lines under these conditions (Figure 4E). Moreover, free PTL and PTL encapsulated in nanoparticles (CMD‐BHQ3‐PTL) exhibited dose‐dependent cytotoxic effects on the two CRC cell lines.

Subsequently, we employed the MTT assay to assess the cytotoxicity of PTL, CMD‐BHQ3, and CMD‐BHQ3‐PTL (pH 7.4 and 5.8) on CRC cells (SW480 and LoVo). The results indicated a dose‐dependent response in the viability of SW480 and LoVo cells after treatment with different concentrations of PTL, CMD‐BHQ3, and CMD‐BHQ3‐PTL (pH 7.4 and 5.8) for 48 h (Figure 4E,F). Notably, under acidic conditions, the inhibitory effect of nanoparticles on tumor cells was most pronounced and closely associated with the acidic environment, promoting drug release. Figure S4A (Supporting Information) presents the specific concentration‐dependent results. We conducted a clonogenic assay to investigate the potential of cell populations with reproductive capacity and to assess the proliferative capability of tumor cells. Under acidic conditions, the number of colonies was significantly lower in the CMD‐BHQ3‐PTL (pH 5.8) treatment group than in the PTL treatment group (*P* < 0.05), indicating that CMD‐BHQ3‐PTL effectively inhibited tumor cell proliferation in an acidic environment (Figure 4G,J). We assessed the inhibitory effects of nanoparticles on the invasion and migration of tumor cells using wound healing and cell invasion assays. Compared to free PTL, CMD‐BHQ3‐PTL (pH 5.8) significantly inhibited the migration of SW480 cells (*P* < 0.05; Figure 4H,J). Additionally, stronger inhibition of cell invasion and migration was observed in the CMD‐BHQ3‐PTL (pH 5.8) treatment group (Figure 4I,J). Regarding cell migration and invasion, we observed that the therapeutic effect of CMD‐BHQ3‐PTL (pH 5.8) was significantly greater than that of PTL. However, both CMD‐BHQ3‐PTL (pH 5.8) and PTL treatment groups exhibited similar inhibition of cell viability, indicating that the primary role of CMD‐BHQ3‐PTL (pH 5.8) and PTL is to suppress tumor cell metastasis. Consequently, the results of migration and invasion studies suggest that CMD‐BHQ3‐PTL (pH 5.8) exhibits a better ability to inhibit invasion and migration due to its sustained release characteristics. These in vitro cytotoxicity and anti‐metastasis experiment results indicated that the CMD‐BHQ3‐PTL (pH 5.8) group was significantly superior to the free PTL group regarding anti‐tumor proliferation, invasion, and metastasis.

We selected SW480 and LoVo cells to induce stemness as their focus and investigate the influence of CMD‐BHQ3‐PTL (pH 5.8) on the stemness of CRC cells. Compared to regular CRC cells, CMD‐BHQ3‐PTL (pH 5.8) demonstrated a significantly reduced toxic effect on tumor cells following stemness induction (*P* < 0.05; Figure 4K). Moreover, compared to CMD‐BHQ3 or PTL, CMD‐BHQ3‐PTL (pH 5.8) effectively suppressed the expression of stemness‐related molecules, including Nanog, Oct‐4, CD44, and CD133 (Figure 4L; Figure S4B, Supporting Information). These results indicated that CMD‐BHQ3‐PTL exerted inhibitory effects on the stemness of CRC cells.

### 2.4 The Novel Nanomaterial CMD‐BHQ3‐PTL/DOX@RBCm Exhibited Superior Inhibitory Effects on Tumor Progression Induced by CRC Stemness

The CMD‐BHQ3‐PTL exhibits outstanding performance in various aspects; however, its relatively low drug‐loading capacity may limit its effectiveness in high‐efficiency treatments.^[^ ^36^, ^37^, ^41^ ^]^ Besides, the absence of specific encapsulation techniques may result in drug leakage or instability, influencing its in vivo behavior.^[^ ^42^, ^43^ ^]^ These challenges emphasize the ongoing necessity for the continuous optimization and enhancement of novel nanomaterials to improve therapeutic outcomes and achieve greater success in cancer treatment. DOX is a conventional chemotherapeutic drug for CRC that can regulate stem cells; however, it may also promote the proliferation and drug resistance of tumor stem cells.^[^ ^44^, ^45^ ^]^ We investigated the synergistic effect of encapsulating DOX in nanomedicine with PTL to target the stemness of CRC cells. Compared to CMD‐BHQ3‐PTL, CMD‐BHQ3‐PTL/DOX@RBCm (Figure 2A,B; Figure S1, Supporting Information) demonstrated significant superiority by combining the excellent performance of the original CMD‐BHQ3‐PTL drug delivery system with the synergistic therapeutic effects of DOX and RBCM encapsulation technology. This comprehensive design provides unique advantages, such as a higher drug payload, enhanced serum stability, and superior targeting capability. These improvements make CMD‐BHQ3‐PTL/DOX@RBCm more effective in inhibiting CRC stemness‐induced tumor progression.

**Figure 5:**
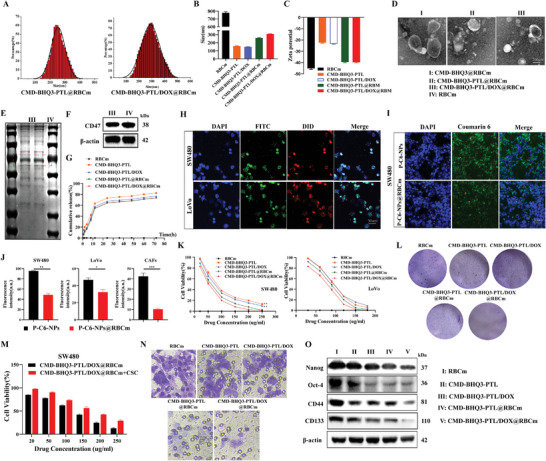
CMD‐BHQ3‐PTL/DOX@RBCM inhibited CRC stem cell‐induced tumor progression. A) Size distributions of CMD‐BHQ3‐PTL@RBCM and CMD‐BHQ3‐PTL/DOX@RBCM measured by DLS(*n* = 3). B) Average particle sizes of RBCM, CMD‐BHQ3‐PTL, CMD‐BHQ3‐PTL/DOX, CMD‐BHQ3‐PTL@RBCm, and CMD‐BHQ3‐PTL/DOX@RBCM in each group(*n* = 3). C) Zeta potentials of the aforementioned groups(*n* = 3). D) TEM images of CMD‐BHQ3@RBCM, CMD‐BHQ3‐PTL@RBCM, and CMD‐BHQ3‐PTL/DOX@RBCM(*n* = 3). E) SDS‐PAGE analysis of red blood cell vesicles (I) and CMD‐BHQ3‐PTL/DOX@RBCM (II) (*n* = 3). F) WB analysis of CD47 in red blood cell vesicles (IV) and CMD‐BHQ3‐PTL/DOX@RBCM (III). G) PTL release in PBS (pH 5.8) for each group. H) Laser confocal observation of SW480 and LoVo cells after incubation with FITC‐labeled CMD‐BHQ3‐PTL/DOX in DiD‐labeled RBCM. Cell nuclei were stained with DAPI (blue), CMD‐BHQ3‐PTL/DOX was labeled with FITC (green), and RBCMs were labeled with DiD (red) (*n* = 3). I) Uptake of P‐C6‐NPs and P‐C6‐NPs@RBCM by SW480 cells(*n* = 3). J) Statistical analysis of nanoparticle uptake in SW480, LoVo, and CAFs cells. K) MTT assay for SW480 and LoVo cells to study the cytotoxicity in each group. L) Colony formation assay results and statistical analysis. M) MTT assay for SW480 cells after co‐intervention with cell stemness culture medium and CMD‐BHQ3‐PTL/DOX@RBCm. N) Cell invasion assay results and statistical analysis. O) WB detection of cell stemness. Statistical results are presented as the mean ± SD using a student's t‐test or one‐way ANOVA. *P < 0.05; **P < 0.01; ***P < 0.001.

DLS measurements revealed that the particle sizes of CMD‐BHQ3‐PTL@RBCm and CMD‐BHQ3‐PTL/DOX@RBCm were 248.22 ± 1.034 and 269.33 ± 0.856 nm, respectively (**Figure** **5**A–C). The zeta potentials for CMD‐BHQ3‐PTL/DOX@RBCM and CMD‐BHQ3‐PTL/DOX@RBCm were −40.23 ± 0.331 and −40.89 ± 0.228 mV, respectively. These data indicated that the introduction of DOX did not significantly alter the particle size and zeta potential of the nanocarriers. Similarly, TEM confirmed that the sizes of CMD‐BHQ3‐PTL@RBCm and CMD‐BHQ3‐PTL/DOX@RBCM were 250 and 270 nm, respectively, consistent with the DLS results (Figure 5D). The increased size was mainly attributed to the successful fusion of nanocarriers with red blood cell vesicles.^[^ ^46^ ^]^ Figure 5E demonstrates that membrane proteins are nearly completely retained on the surface of CMD‐BHQ3‐PTL/DOX@RBCm. Moreover, SDS‐PAGE analysis revealed that CD47 protein expression in CMD‐BHQ3‐PTL/DOX and CMD‐BHQ3‐PTL/DOX@RBCM was nearly identical, indicating the successful encapsulation of RBCM on the CMD‐BHQ3‐PTL/DOX surface (Figure 5F).

Figure 5G demonstrates the in vitro release profile of the nanodrug delivery system at pH 5.8 and 37 °C. PTL was continuously released over 72 h, with a burst release during the initial 10 h. These results indicated a pH‐responsive release pattern for both nanoparticle formulations. Moreover, the drug release rate from non‐encapsulated CMD‐BHQ3‐PTL was faster compared to CMD‐BHQ3‐PTL@RBCM and CMD‐BHQ3‐PTL/DOX@RBCm, without significant difference observed between the latter two. This suggests that coating with RBCM may partially impede drug release. Pre‐stained RBCM were encapsulated using PEG‐FITC‐labeled nanoparticles, and membranes were marked using the red dye DiD. After co‐incubation with tumor cells, the co‐localization of red and green fluorescence signals was observed under confocal microscopy, providing further evidence of the successful encapsulation of RBCM on CMD‐BHQ3‐PTL/DOX nanoparticles (Figure 5H).

Next, we used C6 as a fluorescent dye to track nanoparticle uptake. A brief 4‐h co‐incubation allowed both CRC cells to absorb CMD‐BHQ3‐PTL/DOX (P‐C6‐NPs) or CMD‐BHQ3‐PTL/DOX@RBCm (P‐C6‐NPs@RBCm), emitting green fluorescence, indicating successful penetration of the nanoparticles into the cancer cells (Figure 5I; Figure S5A, Supporting Information). Simultaneously, we investigated the uptake of nanoparticles by CAFs to assess whether P‐C6‐NPs@RBCm influenced fibroblast expression (Figure S5B, Supporting Information). The experimental results confirmed that nanoparticles could be absorbed by CAFs, but the molecular mechanisms involved require further investigation. Figure 5J presents a semi‐quantitative analysis of the above studies. Encapsulation of nanoparticles with RBCM did not significantly affect cellular uptake (*P* > 0.05).

Subsequently, we investigated the therapeutic effects of RBCm, CMD‐BHQ3‐PTL, CMD‐BHQ3‐PTL/DOX, CMD‐BHQ3‐PTL@RBCm, and CMD‐BHQ3‐PTL/DOX@RBCm on SW480 and LoVo cells, focusing on their intervention against CSCs. An MTT assay was conducted to assess cytotoxicity, revealing that SW480 cells exhibited greater sensitivity to the aforementioned treatments than LoVo cells (Figure 5K). Similar outcomes were derived by calculating the IC_50_ of cells (**Table** **1**). Among all the treatment groups, CMD‐BHQ3‐PTL/DOX@RBCm demonstrated a more pronounced inhibition of cancer cell activity and proliferation (Figure 5L; Figure S5C, Supporting Information). Further application of CSC culture medium and simultaneous intervention with CMD‐BHQ3‐PTL/DOX@RBCm on SW480 cells reversed the inhibitory effect on cancer cells post‐CSC intervention (Figure 5M). Additionally, CMD‐BHQ3‐PTL/DOX@RBCm significantly inhibited cancer cell invasion (Figure 5N; Figure S5D, Supporting Information). CMD‐BHQ3‐PTL/DOX@RBCm significantly suppressed the expression of stemness‐related genes, Nanog, Oct‐4, CD133, and CD44 (Figure 5O; Figure S5E, Supporting Information). These results confirmed the inhibitory effect of the novel nanomaterial CMD‐BHQ3‐PTL/DOX@RBCm on the progression induced by CRC stem cells. CMD‐BHQ3‐PTL/DOX and CMD‐BHQ3‐PTL/DOX@RBCm were compared to investigate the influence of RBCM on the pH‐responsiveness of CMD‐BHQ3‐PTL/DOX@RBCm (Figure 5). These results indicated that RBCM did not significantly affect the pH responsiveness of CMD‐BHQ3‐PTL/DOX@RBCm.

**Table 1:** The IC50 of in vitro cytotoxicity in LoVo and SW480 cells for 36h.

IC_50_	LOVO	SW480
PTL	24.36 ± 1.23(µg mL^−1^)	38.26 ± 1.74(µg mL^−1^)
PTL‐NPs	31.20 ± 3.26(µg mL^−1^)	46.81 ± 0.86(µg mL^−1^)
PTL‐NPs@ RBCm	42.91 ± 1.09(µg mL^−1^)	67.94 ± 3.18(µg mL^−1^)

PTL: piceatannol; PTL‐NPs: CMD‐BHQ3‐PTL;

PTL‐NPs@ RBCm:CMD‐BHQ3‐PTL/DOX@RBCm;

The dose‐dependent cytotoxic effects of free PTL, PTL‐NPs, and PTL‐NPs@ RBCm in LoVo cells and SW480 cells for 36 h. Each point presented as mean ± SD (*n* = 3).

### 2.5 CMD‐BHQ3‐PTL/DOX@RBCm Inhibited CRC Stemness via SOX9

**Figure 6:**
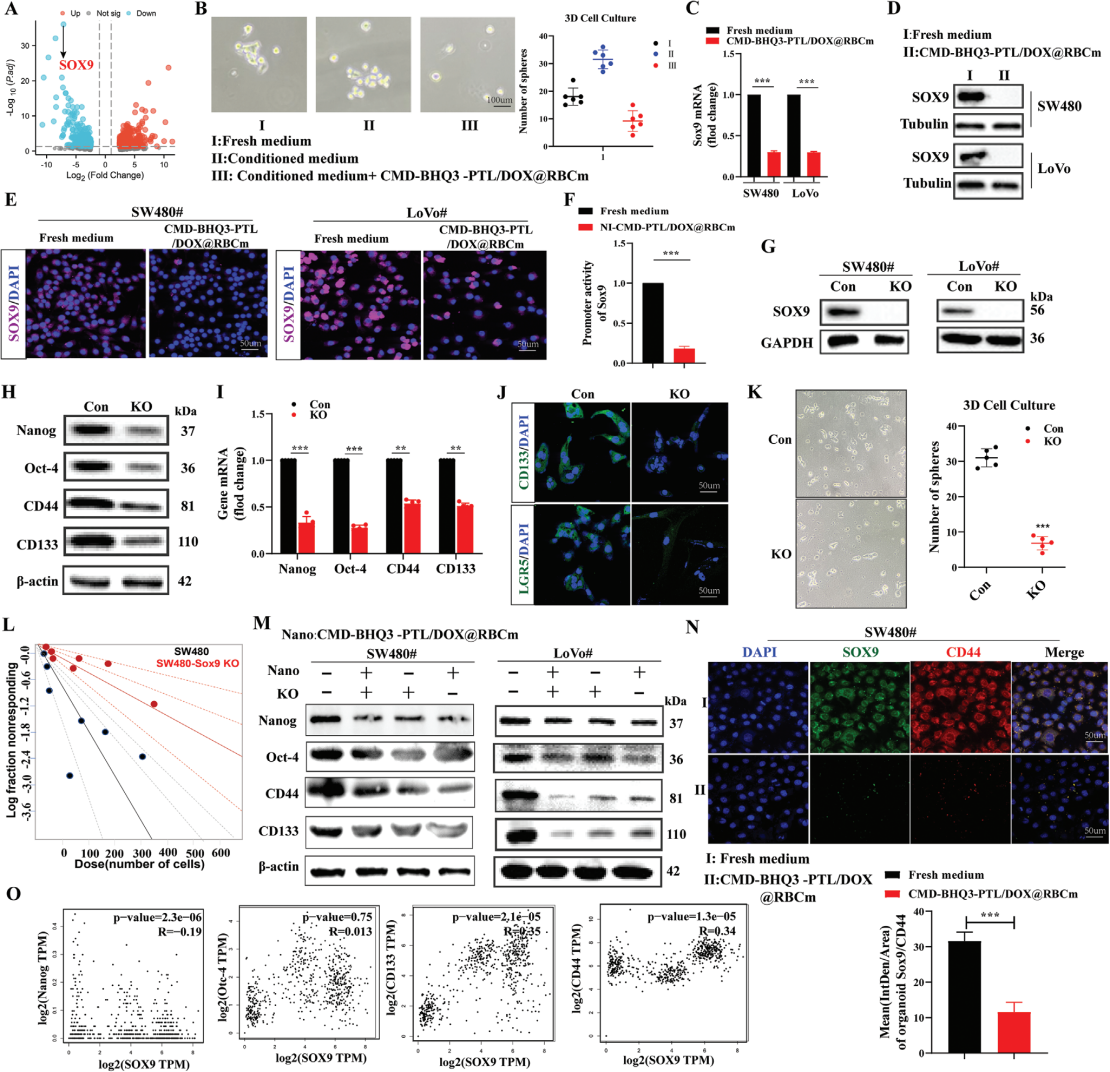
CMD‐BHQ3‐PTL/DOX@RBCm suppressed CRC stemness in SOX9. A) RNA sequencing of CRC treated with culture medium and CMD‐BHQ3‐PTL/DOX@RBCm represented as a volcano plot of DEGs. B) Tumor‐cell pellet experiments. SOX9 expression in CRC cells treated with culture medium and CMD‐BHQ3‐PTL/DOX@RBCm was detected using C) qPCR, D) WB, and E) immunofluorescence staining. F) Luciferase reporter assay evaluating the promoter activity of SOX9 in CRC cells after treatment with CMD‐BHQ3‐PTL/DOX@RBCm. G) WB analysis of SOX9 expression in SW480 and LoVo cells. Stemness‐related gene expression in SOX9‐KO SW480 cells was assessed using H) WB, I) qPCR, J) immunofluorescence, K) sphere formation assay, and L) limiting dilution assay. M) Stemness‐related gene expression in CRC cells after CMD‐BHQ3‐PTL/DOX@RBCm intervention following SOX9‐KO detected using WB. N) Co‐expression of SOX9 and CD44 was observed using confocal microscopy in CRC cells after CMD‐BHQ3‐PTL/DOX@RBCm intervention following SOX9‐KO. O) Verification of the association between SOX9 and stemness‐related genes using limiting dilution experiments. KO: knockout. Con: control. Nano: CMD‐BHQ3‐PTL/DOX@RBCm. Statistical results are presented as the mean ± SD using a student's t‐test or one‐way ANOVA. *P < 0.05; **P < 0.01; ***P < 0.001.

We conducted RNA sequencing to investigate their impact on the transcriptome of CRC cells and elucidate the regulatory mechanism of PTL and its nanoparticles on CRC stemness. Following treatment with CMD‐BHQ3‐PTL/DOX@RBCm, SOX9, a typical member of the SOX family, ranked first among the downregulated differentially expressed genes (DEGs) [Log_2_ (fold change)  = −5.045; DEGs filtering criteria: |Log_2_ (fold change)| > 1, FDR = 0.0016 and *P* < 0.05; **Figure** **6**A]. We collected conditioned medium from CRC cells and cultured CRC cells in this medium. Simultaneously, we intervened with CMD‐BHQ3‐PTL/DOX@RBCm in a 7‐day 3D cell culture to assess the sphere‐forming ability of the cells. The results demonstrated that the conditioned medium significantly enhanced the stemness of CRC cells, while the nanomedicine markedly inhibited cell stemness (Figure 6B). RT‐qPCR (Figure 6C), WB (Figure 6D), and immunofluorescence staining (Figure 6E; Figure S6B, Supporting Information) revealed a significant reduction in SOX9 expression in SW480 and LoVo CRC cells treated with CMD‐BHQ3‐PTL/DOX@RBCm in vitro. Luciferase assays demonstrated significant inhibition of the transcriptional activity of the SOX9 promoter in CRC‐SW480 by CMD‐BHQ3‐PTL/DOX@RBCm (Figure 6F). These results suggested that SOX9 may serve as a downstream target in modulating CRC stemness by CMD‐BHQ3‐PTL/DOX@RBCm.

We constructed lentiviruses with SOX9‐ (KO) and stable transfection into SW480 and LoVo cells. Figure 6G indicates the successful establishment of CRC cells with SOX9‐KO. Further analysis using WB (Figure 6H), qPCR (Figure 6I), immunofluorescence (Figure 6J), sphere formation assays (Figure 6K), and limiting dilution experiments (Figure 6L) revealed significant inhibition of stemness in CRC cells after SOX9‐KO. Subsequently, we intervened in CRC cells with SOX9‐KO using CMD‐BHQ3‐PTL/DOX@RBCm and assessed stemness in tumor cells using WB (Figure 6M; Figure S6E,F, Supporting Information), immunofluorescence experiments (Figure 6N), and correlation analysis (Figure 6O). The results demonstrated a synergistic inhibitory effect of CMD‐BHQ3‐PTL/DOX@RBCm and SOX9‐KO on CRC stemness. In conclusion, CMD‐BHQ3‐PTL/DOX@RBCm overcame CRC stemness via a SOX9‐dependent mechanism.

### 2.6 CMD‐BHQ3‐PTL/DOX@RBCm Decreases SOX9 Expression by Inhibiting the Activation of the Hippo/YAP1 Signaling Pathway in CRC

**Figure 7:**
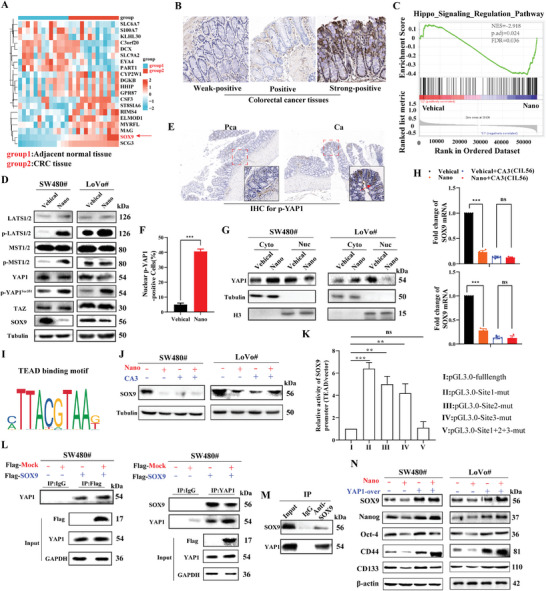
CMD‐BHQ3‐PTL/DOX@RBCm decreased SOX9 expression by inhibiting activation of the Hippo/YAP1 signaling pathway in CRC. A) The GSE222298 dataset from the GEO database revealed the transcriptomic profiles of normal and CRC tissues. B) Immunohistochemistry analysis of SOX9 expression in normal, adjacent, and tumor tissues from patients with CRC. C) RNA sequencing data and GSEA revealing the impact of CMD‐BHQ3‐PTL/DOX@RBCm on gene set enrichment in CRC. D) Immunoblot experiments delineated the molecular mechanisms of CMD‐BHQ3‐PTL/DOX@RBCm on the Hippo/YAP1 signaling pathway. E,F) Immunohistochemistry analysis provides semi‐quantitative results of YAP1 expression in adjacent (pCa) and tumor tissues (Ca). G) Immunoblot experiments to detect YAP1 expression in the nucleus and cytoplasm. H) qPCR was used to assess SOX9 expression in SW480 and LoVo cells following CMD‐BHQ3‐PTL/DOX@RBCm and CA3 interventions. I) JASPAR predicted the potential binding sites in the YAP1 promoter. J) Immunoblot experiments assessing SOX9 expression in SW480 and LoVo cells after CMD‐BHQ3‐PTL/DOX@RBCm and CA3 interventions. K) Dual‐luciferase assays were used to determine the impact of YAP1/TEAD on the transcriptional activity of the SOX9 promoter in CRC cell lines. L) IP experiments revealed the interaction between YAP1 and SOX9 in CRC cells following CMD‐BHQ3‐PTL/DOX@RBCm intervention. M) IP experiments exploring the interaction between YAP1 and SOX9 in CRC tissues after CMD‐BHQ3‐PTL/DOX@RBCm intervention. N) Immunoblot experiments assessing the impact of CMD‐BHQ3‐PTL/DOX@RBCm intervention on the stemness of CRC cells overexpressing YAP1. Statistical results are presented as the mean ± SD using a student's t‐test or one‐way ANOVA. *P < 0.05; **P < 0.01; ***P < 0.001.

After assessing the expression and function of SOX9 in CRC cells, we further analyzed SOX9 expression in normal and CRC tissues using bioinformatic tools. Initially, we observed high SOX9 expression in CRC tissues by comparing the transcriptome sequencing results of CRC patients' adjacent normal and CRC tissues from the GSE222298 dataset in the GEO database (https://www.ncbi.nlm.nih.gov/geo/; **Figure** **7**A). Subsequently, we conducted an analysis using TCGA database (https://portal.gdc.cancer.gov/), UALCAN, and GEPIA and combined information from the Xiantao Academic website to compare the transcriptomes of 134 normal and 134 tumor tissues. The analysis revealed elevated SOX9 expression in CRC tissues (Figure S7A–C, Supporting Information). We found a negative correlation between high SOX9 expression and patient prognosis by analyzing data from GEPIA and the Xiantao Academic website and examining the correlation between SOX9 levels and the survival period of gastric cancer patients (Figure S7D,E, Supporting Information). Furthermore, immunohistochemical analysis of normal, adjacent, and tumor tissues collected from patients with cancer confirmed elevated SOX9 expression in tumor tissues (Figure 7B).

We performed gene set enrichment analysis (GSEA) using RNA sequencing data from CRC treated with culture medium and CMD‐BHQ3‐PTL/DOX@RBCm to investigate the molecular mechanisms underlying the reduced SOX9 expression in CRC by CMD‐BHQ3‐PTL/DOX@RBCm. The Hippo/YAP1 signaling pathway emerged as a top‐enriched pathway (Figure 7C). Given the sensitivity of YAP1 phosphorylation, we assessed the impact of CMD‐BHQ3‐PTL/DOX@RBCm on YAP phosphorylation. However, total YAP1 expression remained unchanged, and phosphorylated YAP (Ser351) was significantly altered. Subsequently, we assessed LATS1 and MST1 phosphorylation, where CMD‐BHQ3‐PTL/DOX@RBCm induced their phosphorylation without changing total levels, akin to its impact on YAP1 (Figure 7D; Figure S7F–I, Supporting Information). Further investigation revealed pronounced upregulation of YAP1 in tumor tissues (Figure 7E,F). Upon phosphorylation by LATS1 and other kinases, YAP1 is sequestered in the cytoplasm and adopts an inactive transcriptional form. Figure 7G demonstrates that CMD‐BHQ3‐PTL/DOX@RBCm treatment reduced nuclear YAP1 levels and elevated cytoplasmic YAP1 levels in CRC cells, indicating inhibition of YAP1 nuclear translocation. In addition, we observed that the inhibitor CA3 (IL56) effectively nullified the influence of CMD‐BHQ3‐PTL/DOX@RBCm on SOX9 expression, suggesting that the reduction in SOX9 expression by CMD‐BHQ3‐PTL/DOX@RBCm was achieved by inhibiting the Hippo/YAP1/TAZ signaling pathway in CRC (Figure 7H,J). As highlighted earlier, the interaction between YAP and the DNA‐binding transcription factor TEAD plays a pivotal role in regulating the activity of the target gene promoters. We identified two potential TEAD‐binding sites within the YAP1 promoter using the online transcription factor prediction software JASPAR (Figure 7I). Subsequent luciferase assays revealed that each TEAD binding site necessitated mutated constructs for complete promoter activity, with the mutation of both sites resulting in complete loss of luciferase activity. This underscores the indispensable role of TEAD in modulating SOX9 promoter activity (Figure 7K).

Subsequently, we investigated the reciprocal interaction between SOX9 and YAP1 at both cellular and tumor tissue levels, employing immunoprecipitation (IP) experiments. Our experimental outcomes unequivocally established a direct interplay between SOX9 and YAP1, evident in both cellular and tissue protein analyses (Figure 7L,M). Further experiments aimed at restoration corroborated that the suppressive impact of CMD‐BHQ3‐PTL/DOX@RBCm on stemness in SW480 and LoVo tumor cells could be counteracted by stable transfection of YAP1 via lentivirus overexpression (Figure 7N). These findings provide compelling evidence that CMD‐BHQ3‐PTL/DOX@RBCm attenuates SOX9 expression by impeding activation of the Hippo/YAP1 signaling pathway in CRC.

### 2.7 CMD‐BHQ3‐PTL/DOX@RBCm Modulated Tumor Stemness via the Hippo/YAP1/SOX9 Pathway to Suppress Tumor Metastasis

**Figure 8:**
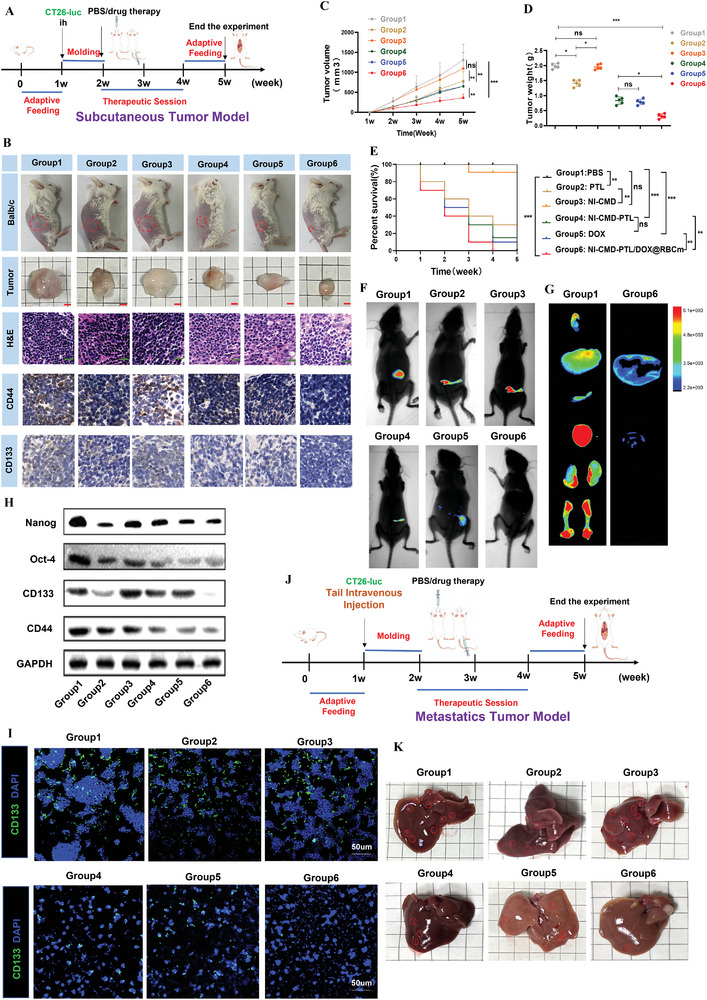
CMD‐BHQ3‐PTL/DOX@RBCM suppressed CRC metastasis by modulating cancer stemness. A) Schematic illustration of subcutaneous tumor model establishment and treatment regimen in mice. B) Macroscopic images of mice from each group, including HE staining and immunohistochemical detection of CD44+ CD133. C) Statistical graph of subcutaneous tumor volumes. D) Statistical graph of subcutaneous tumor masses. E) Survival analysis of mice with subcutaneous tumors in each group. F) In vivo imaging of subcutaneous tumors in mice in each group. G) In vivo organ imaging of mice in the metastasis model, including normal and nanoparticle treatment groups. H) Immunoblotting of tumor stemness in the subcutaneous tumors of mice. I) Laser confocal imaging of the intracellular stemness marker CD133 in CRC cells from various experimental groups. J) Schematic diagram for establishing the mouse metastasis model. K) Results of liver metastasis in mice in the metastatic model. Statistical results are presented as the mean ± SD using a student's t‐test or one‐way ANOVA. *P < 0.05; **P < 0.01; ***P < 0.001.

Next, we established subcutaneous tumors using this scheme (**Figure** **8**A). When the average volume of subcutaneous tumors reached 150 mm^3^, the mice were randomly assigned to six treatment groups (Group 1: PBS; Group 2: PTL, 5 mg kg^−1^; Group 3: CMD‐BHQ3, 5 mg kg^−1^; Group 4: CMD‐BHQ3‐PTL, 5 mg kg^−1^, calculated based on PTL dosage; Group 5: DOX, 20 µg per mouse; and Group 6: CMD‐BHQ3‐PTL/DOX@RBCM, 5 mg kg^−1^, calculated based on PTL dosage). Simultaneously, we conducted a biosafety analysis for each group (Figure S8A, Supporting Information), and the results indicated that the drugs in all treatment groups exhibited no significant biological toxicity.

We conducted a pathological analysis of tumor tissues from different treatment groups. Tumors in Group 6 exhibited significant tumor cell necrosis, while other treatment groups exhibited noticeably fewer necrotic cells in tumor tissues (Figure 8B). Moreover, we performed immunohistochemical staining for the stem cell markers CD44 and CD133 in tumor tissues. The results revealed that Group 6 significantly suppressed stemness in tissues (Figure S8C,D, Supporting Information), with other treatment groups presenting lower stemness inhibition compared to Group 6. Simultaneously, we monitored the volume (Figure 8B,C; Figure S8B, Supporting Information) and mass (Figure 8B,D, Supporting Information) of mouse tumors during the treatment period. The results indicated that tumors in mice treated with PBS and CMD‐BHQ3 grew rapidly, while those in the PTL, CMD‐BHQ3‐PTL, and DOX groups displayed similar tumor inhibition effects, although not significantly. The CMD‐BHQ3‐PTL/DOX@RBCM exhibited a stronger inhibitory effect on mouse tumor growth. Survival analysis of mice from each group revealed that the survival period was longer in the CMD‐BHQ3‐PTL/DOX@RBCM group than in the other model and treatment groups (Figure 8E).

To confirm that this result was not due to the toxicity of PTL or the carrier itself, we examined changes in the structure of mouse organ tissues using HE staining. The results indicated no apparent toxicity of the drugs to mouse organs. The live image analysis of tumor fluorescence in mice confirmed (Figure 8F; Figure S8E, Supporting Information) that the CMD‐BHQ3‐PTL/DOX@RBCM treatment group displayed a significant reduction in vivo fluorescence compared to the model and other treatment groups. This result demonstrated the inhibitory effect of CMD‐BHQ3‐PTL/DOX@RBCM on tumor growth. Moreover, in vivo, CMD‐BHQ3‐PTL/DOX@RBCM significantly inhibited the stemness of mouse tumor tissues (Figure 8H,I; Figure S8F,G, Supporting Information). We conducted a tail vein injection experiment using CT26‐luc cells to further investigate stemness‐related tumor metastasis (Figure 8J). This result confirmed that CMD‐BHQ3‐PTL/DOX@RBCM significantly inhibited circulation‐mediated metastasis of CRC cells (Figure 8G,K; Figure S8H, Supporting Information). These results confirmed that CMD‐BHQ3‐PTL/DOX@RBCM inhibited tumor metastasis by regulating tumor stemness. Statistical results are presented as the mean ± SD using a student's t‐test or one‐way ANOVA. *P < 0.05; **P < 0.01; ***P < 0.001.

**Figure 9:**
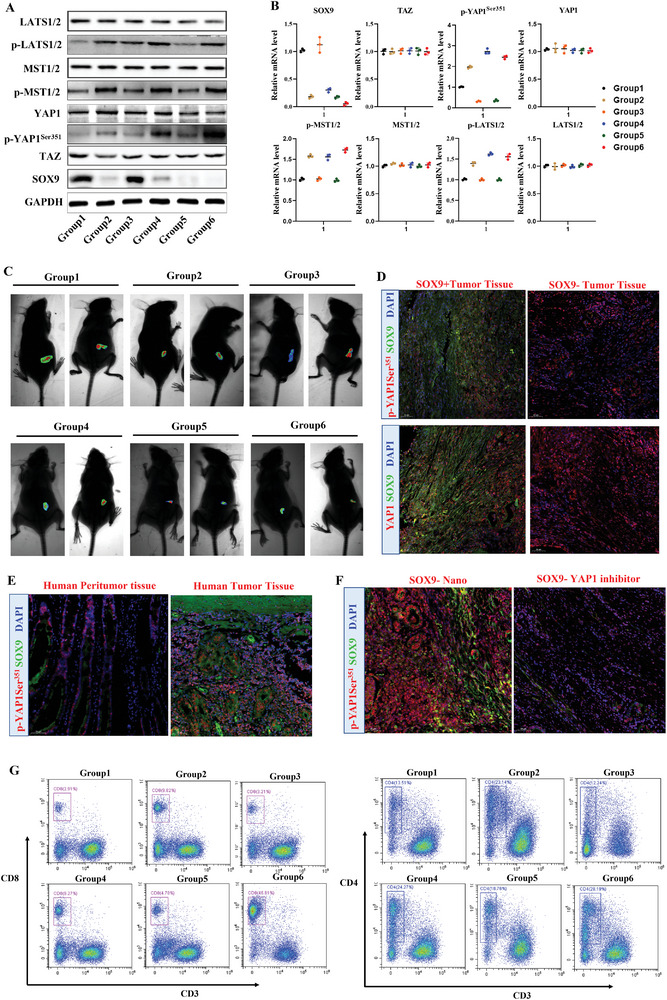
CMD‐BHQ3‐PTL/DOX@RBCM modulated tumor stemness to suppress tumor metastasis via the Hippo/YAP1/SOX9 pathway. A) Immunoblot experiments exploring the regulatory effects of various treatment groups on the Hippo/YAP1/SOX9 pathway. B) qPCR investigating the mechanisms by which different treatment groups modulate the Hippo/YAP1/SOX9 pathway. C) In vivo mouse imaging studies were used to examine the impact of various treatments on tumor progression. D) Immunoco‐localization studies assessing SOX9 expression in subcutaneous tumor tissues, concurrently examining the co‐localization of SOX9 with p‐YAP1 (Ser351). E) Immunoco‐localization of SOX9 and p‐YAP1 (Ser351) co‐expression in adjacent and cancerous tissues in human samples. F) Immunoco‐localization analysis of SOX9 and p‐YAP1 (Ser351) co‐expression in cancer tissues after treatment with the YAP1 inhibitor CA3 (CIL56). G) Flow cytometric analysis of immune infiltration effects of various treatment groups on CD4+ and CD8+ T cells. Statistical results are presented as the mean ± SD using a student's t‐test or one‐way ANOVA. *P < 0.05; **P < 0.01; ***P < 0.001.

Next, we explored the role of Hippo/YAP1/SOX9 pathway in CRC stemness‐related metastasis. We evaluated the impact of CMD‐BHQ3‐PTL/DOX@RBCm treatment on YAP phosphorylation in tumor tissues at the protein and gene expression levels in vivo (**Figure** **9**A,B; Figure S9C, Supporting Information). CMD‐BHQ3‐PTL/DOX@RBCm did not affect the overall YAP1 expression and significantly activated p‐YAP (Ser351) expression. Additionally, CMD‐BHQ3‐PTL/DOX@RBCM markedly induced the phosphorylation of LATS1 and MST1 without altering their total levels of LATS1 and MST1. Meanwhile, CMD‐BHQ3‐PTL/DOX@RBCm distinctly suppressed SOX9 expression. We designed a subcutaneous tumor model by knocking out SOX9 in CRC cells to further validate the molecular mechanisms of CMD‐BHQ3‐PTL/DOX@RBCm (Figure S9A, Supporting Information). Live imaging results in mice confirmed that SOX9‐KO exhibited a tumor inhibitory effect similar to that of CMD‐BHQ3‐PTL/DOX@RBCm (Figure 9C; Figure S9B, Supporting Information). This further demonstrated that SOX9 is a regulatory target of CMD‐BHQ3‐PTL/DOX@RBCm at the tissue level. Immunofluorescence co‐localization experiments compared the co‐expression of SOX9 and p‐YAP1 (Ser351) in subcutaneous tumor tissues with SOX9 overexpression and SOX9‐KO (Figure 9D) and in human tissues between adjacent and cancerous tissues (Figure 9E). These results confirmed a distinct correlation between the expression of p‐YAP1 (Ser351) and SOX9 in mouse and human tissues. Next, we applied CMD‐BHQ3‐PTL/DOX@RBCm in combination with the YAP1 inhibitor CA3 (CIL56) (Figure 9F). These results demonstrated that the effect of CMD‐BHQ3‐PTL/DOX@RBCm was significantly inhibited after YAP1 suppression, proving that the Hippo/YAP1 pathway is a direct downstream regulatory pathway of CMD‐BHQ3‐PTL/DOX@RBCm.

As immune regulation plays a crucial role in the Hippo/YAP1 pathway's control of CRC metastasis, we further conducted adaptive immune‐related flow cytometry detection using the gating strategy (Figure S9D, Supporting Information). Flow cytometry results confirmed that CMD‐BHQ3‐PTL/DOX@RBCm promoted the immune infiltration of CD4+ and CD8+ T cells in mice (Figure 9G). These results indicate that T cell‐related immune activation may be a critical regulatory mechanism of the Hippo/YAP1/SOX9 pathway in CRC stemness‐related metastasis, providing a key direction for future research. In summary, CMD‐BHQ3‐PTL/DOX@RBCm inhibits CRC stemness‐related metastasis via the Hippo/YAP1/SOX9 pathway.

## 3 Discussion

In this study, we investigated the potential mechanisms and efficacy of a novel nanomedicine, CMD‐BHQ3‐PTL/DOX@RBCm, in treating CRC. Systematic experimental design and cellular analyses provided insights into the inhibitory effects of this nanomedicine on CRC growth and metastasis. For the first time, our findings demonstrated the significant inhibitory effects of CMD‐BHQ3‐PTL/DOX@RBCm on CRC. The nanomedicine exhibited outstanding anti‐tumor activity, superior cellular uptake, drug release properties, and modulation of the Hippo/YAP1/SOX9 pathway.^[^ ^47^ ^]^ These findings reinforce the potential therapeutic value of this innovative nanomedicine. The innovation of this study lies in integrating various therapeutic strategies, such as drug synergism and RBCm encapsulation technology. CMD‐BHQ3‐PTL/DOX@RBCm efficiently inhibited CRC cells in vitro and demonstrated remarkable anti‐tumor effects in vivo, providing novel insights for nanomedicine optimization and deepening our understanding of the role of the Hippo/YAP1/SOX9 pathway in CRC development.^[^ ^48^, ^49^ ^]^

A pivotal aspect of our investigation was the outstanding efficacy of CMD‐BHQ3‐PTL/DOX@RBCm in treating CRC. This novel nano‐delivery system demonstrated a level of effectiveness that distinguished it from prior research endeavors. Notably, its exceptional performance in facilitating local drug delivery is a key departure from conventional studies.^[^ ^49^ ^]^ The nano‐delivery system demonstrated superior capabilities, ensuring efficient penetration and targeted release of PTL/DOX, thereby significantly enhancing the therapeutic outcomes. Moreover, the specificity exhibited by CMD‐BHQ3‐PTL/DOX@RBCm in targeting tumor stem cells marks a substantial advancement compared to existing studies. The capacity of the nano‐delivery system to selectively locate these cells and exert its therapeutic effects represents a significant breakthrough.^[^ ^32^ ^]^ This specificity is critical in suppressing stemness‐related processes, slowing tumor progression, and potentially offering a more nuanced and effective treatment approach for CRC.^[^ ^50^ ^]^ Another distinctive feature of CMD‐BHQ3‐PTL/DOX@RBCm was its ability to downregulate SOX9 and induce YAP1 phosphorylation. These molecular mechanisms play pivotal roles in attenuating the proliferative, invasive, and migratory capacities of stem cells within the tumor microenvironment.^[^ ^25^ ^]^ These intricate regulatory actions contribute to the enhanced effectiveness of CMD‐BHQ3‐PTL/DOX@RBCm and a better understanding of the complex interplay between stem cells and the metastatic processes associated with CRC.

Despite limitations, such as potential biases and reliance on in vitro and mouse models, we suggest future research directions. Diverse in vitro models, comprehensive animal models, and monitoring of biological behavior in the human body can address these limitations, allowing for a more personalized approach to anti‐tumor treatment.^[^ ^2^ ^]^ CMD‐BHQ3‐PTL/DOX@RBCm demonstrated potential clinical application value, offering a new avenue for CRC therapy. Optimizing the delivery system, increasing the drug payload capacity, enhancing targeting specificity, and investigating molecular mechanisms in future research may contribute to a comprehensive understanding and foster innovation in the field.^[^ ^5^ ^]^

CMD‐BHQ3‐PTL/DOX@RBCm, a novel nano‐delivery system, holds significant promise for treating CRC. Experimental validation consistently supports its outstanding therapeutic effects, extending beyond nanomedicine design and preparation. CMD‐BHQ3‐PTL/DOX@RBCm's potential clinical application underscores the need for further research, encouraging the medical community to focus on its development and optimization for more effective and innovative treatment options for CRC.

## 4 Experimental Section

### Materials and Cell Culture

PTL ≥ 98.0%, Coumarin‐6 (C6 ≥ 98.0%), and nanomedicine synthesis reagents were procured from Aladdin Biochemical Technology Co. Ltd. (Shanghai, China). RIPA lysis buffer and skim milk powder were obtained from Epizyme (Shanghai, China). RPMI 1640 and fetal bovine serum (FBS) were supplied by Biological Industries (Israel). Human CRC cell lines, HT29 and HCT116, were sourced from the National Authentication Cell Culture Collection and cultured in RPMI 1640 with 10% FBS and 1% penicillin/streptomycin.

### Drug Loading Content (DLC) and Encapsulation Efficiency (EE)

High‐performance liquid chromatography (HPLC) was employed to measure the DLC and EE of PTL using a Shimadzu LC‐10AD instrument (Shimadzu, Japan). The mobile phase for HPLC consisted of methanol/distilled water (60/40, v/v) with a flow rate of 1.0 mL min^−1^ and a detection wavelength of 306 nm. DLC and EE were calculated based on HPLC data.

### Colony Formation Assay

CRC cells were seeded in a six‐well plate at a density of 800 cells per well and treated with drugs during the growth period for 48 h. After treatment, the culture medium containing drugs was replaced with fresh RPMI 1640, and cells were incubated for two weeks, with the medium changing every three days. After 14 days, the cells formed colonies and were fixed with 4% paraformaldehyde. Subsequently, 0.5% crystal violet solution was used for staining, and the results were captured using a camera. After each treatment, the colony numbers were calculated for statistical analysis.

### Sample Synthesis—Synthesis of Self‐Assembled Hypoxia‐Responsive CMD Nanoparticles (HR‐CMD‐NPs)

The synthesis involved coupling the derivative of 2‐nitroimidazole (NI) to the water‐soluble CMD sodium salt (CM‐Dex) backbone through amide formation. Initially, NI was converted to 6‐(2‐nitroimidazole)hexylamine, which was reacted with the carboxylic acid of CM‐Dex. The NI (0.6 g, 5.3 mmol) was dissolved in dimethylformamide (DMF) and reacted with K2CO3 (1.1 g, 7.95 mmol). Subsequently, a DMF solution of 6‐(Boc‐amino)hexyl bromide (1.56 g, 5.57 mmol) was added, and the mixture was stirred overnight at room temperature. The reaction mixture was filtered and washed with methanol, and the solvent was evaporated to yield the NI derivative. Then, NI derivative (0.19–0.573 g, 0.9–2.7 mmol) was coupled to CM‐Dex (0.2 g, 0.9 mmol) in DMF using EDC (0.6–2.07 g, 3.6–10.88 mmol) and NHS (0.41–1.24 g, 3.6–10.88 mmol) as catalysts, and stirred for 1 day. After dialysis, distilled water dialysis, and freeze‐drying, the quantity of NI derivative on CM‐Dex was determined using ultraviolet‐visible (UV–vis) spectrophotometry at the characteristic peak of the NI derivative at 325 nm.

### Sample Synthesis—Synthesis of CMD‐BHQ3‐PTL/DOX@RBCm

Building on the aforementioned approach, more hypoxia‐responsive nanoparticles (HR‐NPs), CMD‐BHQ3, were prepared by chemically modifying CMD with BHQ3. CMD (0.9 mmol) was dissolved in a mixture of formamide and DMF and stirred for 30 min. Then, EDC (0.88 mmol) and HOBt (0.88 mmol) were added, and the mixture was stirred for 30 min. DMF solution of BHQ3 amine (1.8 mmol) was added and stirred for 24 h. The resulting solution was dialyzed with excess distilled water/methanol (1/3–1/1 v/v) for 1 day, followed by distilled water dialysis for 2 days, and then freeze‐dried. In a tumor‐hypoxic environment, the azo‐benzene derivative was reduced to an aniline derivative through an electron transfer process. Anticancer drugs, PTL and DOX, were physically loaded into the hydrophobic core of the nanoparticles to form CMD‐BHQ3‐PTL/DOX, followed by wrapping a layer of RBCM using a physical extrusion method, resulting in CMD‐BHQ3‐PTL/DOX@RBCm.

### Sample Synthesis—Characterization

The morphology of the nanoparticles was observed using scanning electron microscopy (SEM, TESCAN MIRA LMS, Czech Republic) and transmission electron microscopy (TEM, FEI Talos F200X G2, USA). The particle size was analyzed using a dynamic light scattering (DLS) instrument (Zetasizer Nano ZS‐90, Malvern, UK), and the zeta potential of the nanoparticles was evaluated using a Malvern Nano ZS ZEN3600. The crystal structure and crystallographic properties were analyzed using a powder X‐ray diffractometer (PXRD, United States). Fluorescence spectroscopy data were obtained using a fluorescence spectrophotometer (FLS1000, Edinburgh Instruments, Livingston, UK), and spectral measurements were conducted using a confocal Raman micro‐spectrometer (LabRAM HR Evolution; Kyoto, Japan). X‐ray photoelectron spectroscopy (XPS) spectra were obtained using an XPS spectrometer (ESCALAB 250Xi; Thermo Fisher Scientific, Waltham, MA). UV‐vis‐NIR spectroscopy was conducted using a spectrophotometer (UV‐3600; Shimadzu, Japan).

### In Vitro Release Experiment

The required number of Franz diffusion cells was prepared, and the PBS buffer was configured by adjusting it to the appropriate pH. The nanodrug samples were thoroughly suspended in PBS, and the temperature and humidity were set before the experiment to simulate the human body temperature environment. The experimental instrument was started to ensure the samples were adequately exposed to PBS. At predetermined time points, a certain volume of release fluid was withdrawn, and the withdrawn volume was replaced with fresh PBS to maintain the stability of the release environment. The drug concentration in the release fluid was measured using HPLC or other applicable analytical methods, and drug release was recorded at each time point. Further parameters, such as the release rate and half‐life, were calculated to comprehensively assess the release performance of the nanodrug in an extracellular environment.

### Western Blotting Experiments(WB)

The process of extracting proteins from tissues or cells involves the use of RIPA buffer to disrupt cell membranes and release proteins. Subsequently, the extracted proteins were concentrated through centrifugation to obtain a sample of sufficient concentration. Protein quantification was carried out using the Bradford method to ensure equal loading of protein amounts. Next, the proteins are mixed and heated, followed by separation using SDS‐PAGE. Upon completion of separation, the proteins were transferred onto a PVDF membrane, facilitated by an electric field, forming protein bands identical to those on the gel. To prevent antibody binding to nonspecific sites, bovine serum albumin (BSA) was employed as a blocking agent, followed by the addition of primary antibodies and overnight incubation at 4 °C. Excess primary antibodies were removed through washing steps to reduce nonspecific binding. Subsequently, secondary antibodies from a different host species than the primary antibodies were introduced, followed by another round of washing to eliminate unbound secondary antibodies. Finally, a fluorescence imaging system or chemiluminescence system was utilized to detect the markers on the secondary antibodies, allowing observation of the position and relative intensity of protein bands. Quantitative analysis of experimental results was performed using image analysis software or related instruments to assess the expression levels of the target proteins.

### MTT Assay

The MTT (3‐(4,5‐dimethylthiazol‐2‐yl)‐2,5‐diphenyltetrazolium bromide) assay was employed to assess cell viability under different treatments. Cells were seeded at an appropriate density in a 96‐well plate and allowed to adhere overnight in the culture medium. Following treatments for 24, 48, and 72 h, MTT solution was added to each well, and cells were incubated to form formazan crystals. After removing the culture medium, DMSO was added to dissolve the crystals, and the absorbance at a specific wavelength was measured using a microplate reader to quantitatively evaluate cell viability. The experiment was independently repeated three times.

### RT‐qPCR Experiment

Total RNA was extracted from processed cells or tissues using the appropriate RNA extraction reagent, ensuring the integrity and purity of the extraction steps according to the manufacturer's instructions. The concentration and purity of RNA samples were assessed using a UV spectrophotometer, ensuring an A260/A280 ratio between 1.8 and 2.0. Subsequently, RNA was reverse transcribed into cDNA using reverse transcriptase, ensuring sufficient reaction conditions and time to obtain high‐quality cDNA. qPCR was performed using suitable fluorescent dyes such as fluorescent probes or SYBR Green, selecting primer pairs matching the research target (**Table** **2**), and running PCR reactions at the appropriate temperature. The size and purity of PCR products were assessed through gel electrophoresis or other methods. Analysis of PCR amplification curves using specific software allowed for the calculation of the relative gene expression levels. Differences in gene expression levels between different groups were compared, and the biological significance of the experimental results was discussed. The entire experiment was independently repeated three times to ensure the reliability and stability of the results.

**Table 2:** The primer sequences of genes in RT‐qPCR experiment.

Gene	Forward sequence	Reverse sequence
CD133	5′‐GGGATCAGCTCCTGGAAAG‐3′	5′‐GGTAGTTGCTGGGGAAGGA‐3′
CD44	5′‐TGCTTGTGGAATTTGGGAGG‐3′	5′‐CCTGAAGACAGCTTGGGGT‐3′
Oct‐4	5′‐GGAGAAGGAGAAGCTGGAG‐3′	5′‐GGTGGTCTAGGTTGGGAAG‐3′
Nanog	5′‐CTTCCTGGCCCTGTCTCTCT‐3′	5′‐GCCAGCTGTTGTTGCAGTGT‐3′
SOX9	5′‐TACCCGGAGCTGGGAAAATA‐3′	5′‐GCACTGGTGTGTGATGAGG‐3′
TAZ	5′‐GCAAGAGCGGAAAGAGTCG‐3′	5′‐CAGGGTGGTGGTAGAGGTT‐3′
p‐YAP1Ser^351^	5′‐TGCTCGCCCTTTATTGGAG‐3′	5′‐CGTGTTGTTGCTGCTTGAA‐3′
YAP1	5′‐ACAACTCGTCCATGGACCTC‐3′	5′‐TGGTCATGCTCTGCTTCACC‐3′
p‐MST1/2	5′‐GAAGACCTTTGGTGGAGAG‐3′	5′‐GAGGTGGCTGAGGATGTAG‐3′
MST1/2	5′‐CTGAAGGTGTGGAGGAGGA‐3′	5′‐TCCTCCAGCCTCTTCTTCCT‐3′
p‐LATS1/2	5′‐AGGCTGTGCTGTGGATGTTG‐3′	5′‐GGCTCCTTTCCCTTGTCTTG‐3′
LATS1/2	5′‐GTGGCTGTGTGGAAGAGA‐3′	5′‐CGGTAGGAAGAGGAAGGA‐3′

### Immunofluorescence Experiments

Sample collection and processing were essential to ensure their suitability for subsequent experiments. Antigen retrieval on fixed tissue samples was performed through heat induction or enzymatic methods, preserving the natural structure of proteins. Enhancing antibody penetration efficiency, 0.1% Triton X‐100 permeabilizes cell membranes. Non‐specific binding sites were blocked using 5% BSA to minimize interference from background signals. Subsequently, specific primary antibodies were introduced, facilitating overnight incubation to enhance binding efficiency with target proteins. Washing steps with buffer solutions or PBS are conducted to remove unbound primary antibodies, ensuring the accuracy of experimental results. Fluorescently labeled secondary antibodies were then added, followed by additional washes to eliminate any unbound secondary antibodies. Staining of cell nuclei with a dye such as DAPI was carried out, and samples were sealed with mounting medium on glass slides to maintain stability. Finally, observation of samples under a fluorescence microscope or laser confocal microscope allows the acquisition of fluorescence images at the corresponding wavelengths, providing crucial insights into the functionality and localization of proteins in biological processes.

### Cell Migration Experiment

Cells were seeded at an appropriate density in sterile culture dishes to maintain active growth. Prior to experimentation, cells underwent pretreatment, exposed to specific stimuli, drugs, or other interventions to simulate physiological or pathological conditions. Transwell chambers were assembled, with the upper chamber having cell culture medium and the lower chamber containing medium with a chemotactic inducer for cell migration. The chambers were placed in a cell culture incubator for a specific period to facilitate migration. After cultivation, cells in the upper chamber were fixed onto the pores at the bottom by rinsing with sterile PBS. Crystal Violet stained the pores, enabling observation and counting of migrated cells. Microscopic examination and image capture at different time points dynamically monitored the cell migration process.

### Cell Invasion Assay

Cultivate cells at an appropriate density in a culture dish, ensuring stable growth. Perform pre‐treatment based on experimental requirements. Assemble the experimental setup with Matrigel‐coated Transwell chambers mimicking the invasion microenvironment. The upper layer contains cell culture medium, while the lower layer had medium with chemical inducers guiding invasion. Add cell suspension to the upper layer, incubate in specific conditions to facilitate penetration. After cultivation, fix upper chamber cells onto the matrix and membrane at the bottom, washing away upper layer cells. Stain bottom chamber cells with crystal violet for observation and counting of invaded cells. Observe under a microscope, capturing images at different time points to monitor invasion dynamically.

### Transmission Electron Microscope Observation Experiment

After collecting cell or tissue samples, wash them with a buffer solution and cut the tissue into small pieces for convenient transmission electron microscopy (TEM) observation. Fix the cells or tissue in TEM fixative at room temperature or 4 °C. Dehydrate the samples using a series of progressively concentrated ethanol solutions, followed by embedding in a medium such as transparent resin to form a fixed block. Use a blade to section the fixed block into thin slices of 70–100 nanometers in thickness, and place them on TEM grids. Enhance image contrast through heavy metal salt staining and counterstaining. Finally, observe the ultrastructure of the samples using transmission electron microscopy, capturing high‐resolution images to delve into microscopic details.

### Sphere Formation Assay

CRC cells (5000 cells mL^−1^) were seeded in serum‐free medium in ultra‐low attachment six‐well plates (Corning, catalog number CLS4520). After two weeks of cultivation, the number of tumor‐spheres with diameters > 75 µm was quantified.

### Mouse Tumor Model

CRC cells were cultured and maintained under sterile conditions until they reached 80% density. After collecting the cells with trypsin, they were suspended in a suitable culture medium. C57BL/6 mice (20 ± 2 g, approximately eight‐week‐old) were acclimatized to the SPF‐grade facilities. Pre‐surgical preparations included shaving, disinfection, and careful anesthesia administration to minimize pain. Then, treated cell suspensions were administered subcutaneously or via tail vein injection using a micro‐syringe. Post‐surgery, vigilant monitoring was implemented to ensure that mice gradually recovered consciousness. Regular observations were conducted to monitor tumor development and record growth parameters. The experiment was concluded at predetermined time points or upon reaching the tumor volume. The relevant tumor tissues were collected for subsequent histological and molecular analyses. This experimental design followed animal ethics guidelines, including Animal Research: Reporting In Vivo Experiments (ARRIVE) and EU Directive 2010/63/EU. This study was approved by the Institutional Animal Care and Use Committee (IACUC) of the United States National Institutes of Health (NIH) under ethics approval number HKTJCT2022‐788.

### Ethics Approval and Consent to Participate

The studies involving animals were reviewed and approved by the Animal Ethics Committee of Tongji Medical College, Huazhong University of Science and Technology (No.2023‐02‐41). The animal experiments were conducted in strict compliance with the animal ethics guidelines set forth by the IACUC of the United States NIH, adhering to the ARRIVE guidelines and EU Directive 2010/63/EU for animal experiments. Animal experiments were executed in strict accordance with protocols sanctioned by the China Pharmaceutical University's Institutional Animal Care and Use Committee (Permit:HUST2023‐10‐114).

### Wound Healing Assay

Cells were cultured at an optimal density in a suitable medium. Through aseptic techniques, cells were transferred to reach 80–90% confluence in culture dishes. Using a cell scraper, a consistent straight‐line scratch was made in the dishes. After gently washing with growth medium or PBS, an appropriate amount of culture medium was added to support cell growth and migration. Microscopic images were taken of the initial scratch to record cellular morphology. The dishes were then returned to the incubator, and the process of cell migration was regularly observed and documented. Microscopic images of the scratched area were captured at different time points for dynamic monitoring of cell migration.

### Statistical Analysis

The data were statistically analyzed using GraphPad Prism software (version 8.2). Descriptive statistics were presented as mean ± standard deviation (SD). Group comparisons for variables conforming to a normal distribution were evaluated using one‐way analysis of variance. Subsequent post‐hoc analysis was conducted using the least significant difference method for pairwise mean distinctions. A significance threshold of *P* < 0.05 was applied to establish statistical significance.

## Conflict of Interest

The authors declare no competing interests.

## Author Contributions

M.Z., H.N., G.H., and M.Z. contributed equally to this work. M.Z., H.N., G.H. performed methodology, investigation, data curation, wrote the original draft, and wrote, reviewed, and edited the draft. D.C., H.L., X.X. performed validation, investigation, and wrote the original draft. H.W., H.Z., F.L., and R.C. performed review and editing, project administration, supervision, formal analysis, and funding acquisition. Manuscript is approved by all authors for publication.

## Supporting information



Supporting Information

## Data Availability

The data that support the findings of this study are available from the corresponding author upon reasonable request.
