# Supplementary material for: Biomimetic Nano‐delivery of Small‐Molecule Piceatannol Modulates Tumor Stemness and Suppresses Colorectal Cancer Metastasis via Hippo/YAP1/SOX9 Signaling
Source: Small. 2024 Nov 10;21(2):2407191. doi: 10.1002/smll.202407191 (PMC11735875; doi:10.1002/smll.202407191)

**Supplementary Materials**

**Material synthesis specification**

**Effect of Black hole quencher 3**

In CMD-BHQ3-PTL/DOX@RBCm, Black hole quencher 3 (BHQ3) plays a pivotal role as a crucial component of the nano-delivery system. As depicted in **Figures 2A** and **2B**, BHQ3 acts as a linker between Piceatannol (PTL)/DOX and carboxymethyl dextran (CMD), facilitating their encapsulation within red blood cell membranes (RBCm). Incorporating BHQ3 into CMD-BHQ3-PTL/DOX@RBCm offers several advantages: **(1)**Enhanced stability: BHQ3 effectively prevents non-specific interactions between PTL/DOX and CMD, thereby stabilizing the nano-delivery system and maintaining its integrity during storage and transmission. (2)Improved biocompatibility: BHQ3 encapsulates PTL/DOX within the nano-delivery system, reducing their potential cytotoxicity and enhancing their biocompatibility and safety within the biological system. (3)Facilitated delivery: BHQ3 serves as a linker, facilitating the encapsulation of PTL/DOX within CMD-based nano-delivery systems, facilitating their targeted delivery to colorectal cancer cells. Incorporating BHQ3 into CMD-BHQ3-PTL/DOX@RBCm not only enables more effective targeted delivery of PTL/DOX but also underscores its importance in drug transportation and therapy.

**pH responsiveness of CMD-BHQ3-PTL/DOX@RBCm**

The pH responsiveness of CMD-BHQ3-PTL/DOX@RBCm is closely associated with the crucial role of tertiary amines. The pH responsiveness of this system is primarily attributed to the tertiary amine groups in CMD. Under physiological pH conditions (approximately 7.4), the tertiary amine groups in CMD-BHQ3-PTL/DOX@RBCm exhibit a higher degree of ionization, resulting in a negatively charged nanodelivery system. When the environment becomes acidic (such as when the pH drops to approximately 6.5-6.8 in tumor tissues), the ionization of the tertiary amine groups decreases, reducing the negative charge. This causes the surface charge of CMD-BHQ3-PTL/DOX@RBCm to transition to neutral or positive, leading to the dissolution or disassembly of the nanoparticles and the release of PTL/DOX. Therefore, tertiary amines play a key role in regulating the pH responsiveness of CMD-BHQ3-PTL/DOX@RBCm, facilitating targeted drug release.

**Statistical results of drug loading efficiency (DLE) and drug loading content (DLC)**

**Supplementary Table S1** Drug Loading capacity (DLC%) of NI-CMD-PTL obtained by incubation of NI-CMD and PTL at different mass ratios And Drug loading efficiency (DLE%).

| **NI-CMD/PTL**  **(mass ratio)** | **Drug Loading capacity (%)** | **Drug loading efficiency(%)** |
| --- | --- | --- |
| 8/1 | 14.5 | 100 |
| 8/2 | 18.9 | 91.5 |
| 8/4 | 25.8 | 88.4 |
| 8/6 | 26.3 | 82.6 |
| 8/8 | 27.2 | 76.1 |
| 8/10 | 29.3 | 69.5 |
| 8/12 | 31.4 | 66.2 |
| 8/14 | 36.1 | 65.7 |
| 8/16 | 24.8 | 42.3 |
| 8/18 | 21.1 | 27.8 |
| 8/20 | 16.8 | 11.3 |

**Advantages of CD-BHQ3 as a carrier for internal delivery of DOX and PTL**

In this study, the main advantages of using CD-BHQ3 as the carrier for internal delivery of DOX and PTL include its pH responsiveness, allowing drug release in the low pH tumor microenvironment, enhancing targeting and efficacy while minimizing adverse effects on normal tissues. Additionally, CD-BHQ3 enables dual drug delivery, optimizing synergistic effects and efficacy. Its biocompatibility and low toxicity contribute to reduced adverse reactions, and the stability of the nano-delivery system is further enhanced by encapsulation within red blood cell membranes (RBCm), prolonging circulation time and increasing drug concentration. Despite the potential for other materials to achieve drug delivery under low oxygen and pH conditions, the unique properties of CD-BHQ3 make it an ideal choice in this study.

**Reasons for choosing picetanol among many mature anti-tumor chemotherapy drugs**

The selection of Piceatannol as an anti-cancer chemotherapy agent is based on several factors. Firstly, Piceatannol is a naturally occurring flavonoid compound with various biological activities, including antioxidant, anti-inflammatory, and anti-cancer properties. Secondly, Piceatannol has been extensively studied and demonstrated anti-tumor effects against various cancer types, including colorectal cancer, both in vitro and in vivo. Additionally, compared to similar compounds, Piceatannol exhibits stable metabolism in the body and has lower toxicity. Lastly, Piceatannol has the ability to modulate the activity of tumor stem cells and inhibit the migration and invasion of tumor cells, making it a potential candidate for cancer therapy.


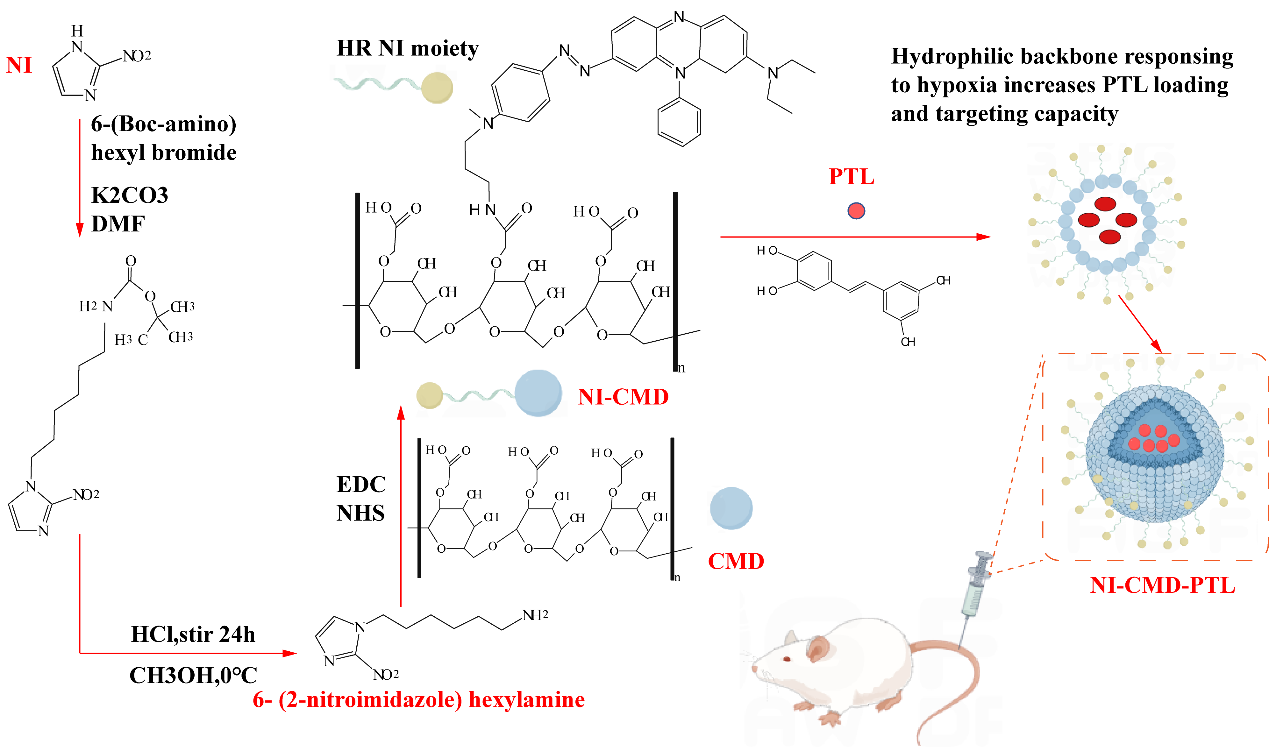


**Figure S1**. **Synthesis of self-assembled HR-CMD-NPs.**


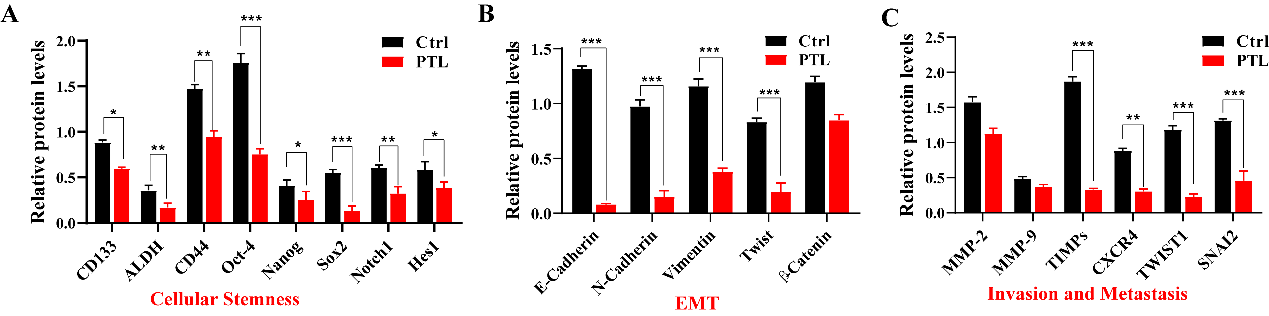


**Figure S2**. **The central mechanism underlying PTL-induced CRC cell apoptosis is the inhibition of cellular stemness.** WB validation presents the correlation of PTL with (**A**) cellular stemness, (**B**) EMT, and (**C**) invasion and metastasis. Grayscale intensity statistical analysis of WB results. Statistical results are presented as the mean ± SD using a student’s t-test or one-way ANOVA. * P < 0.05; ** P < 0.01; *** P < 0.001.


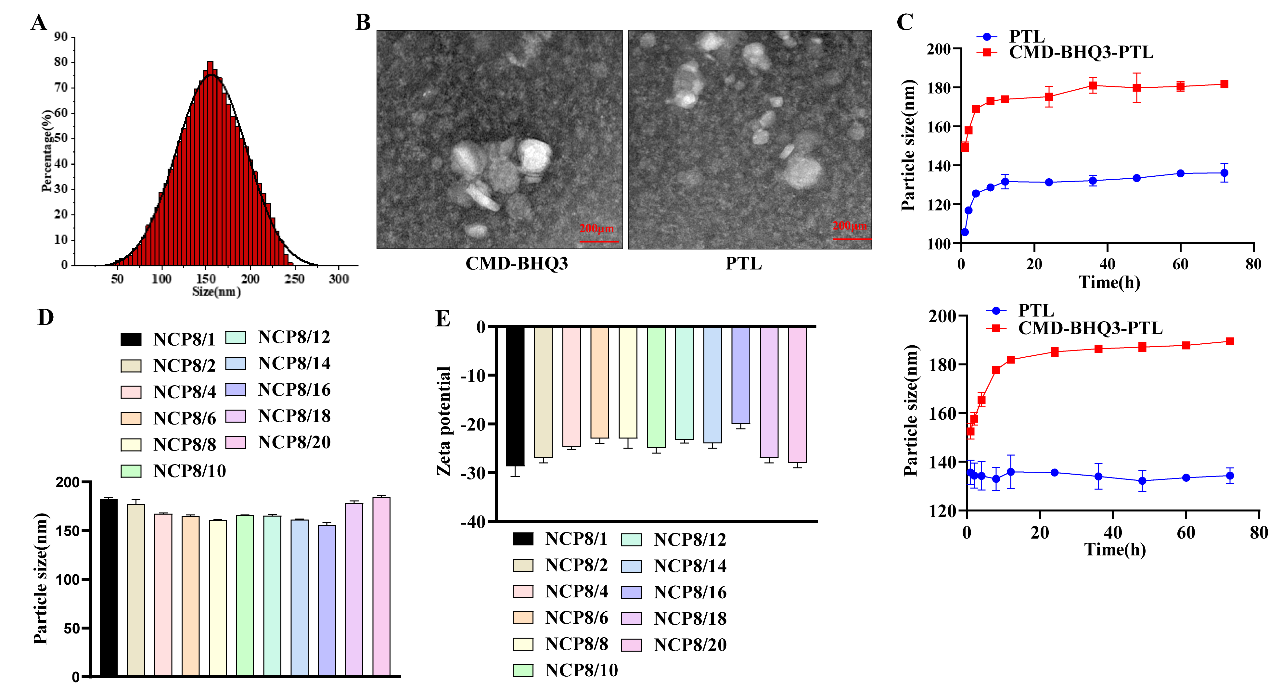


**Figure S3**. **Preparation and characterization of nanoparticles. (A)** Size distribution of CMD-BHQ3, as measured by DLS**.** (**B**) TEM images of CMD-BHQ3 and PTL. (**C**) Stability testing of CMD-BHQ3-PTL *in vitro* in PBS and FBS. Detection of (**D**) particle size and (**E**) zeta potential of synthesized nanoparticles at different feed ratios. Statistical results are presented as the mean ± SD using a student’s t-test or one-way ANOVA. * P < 0.05; ** P < 0.01; *** P < 0.001.


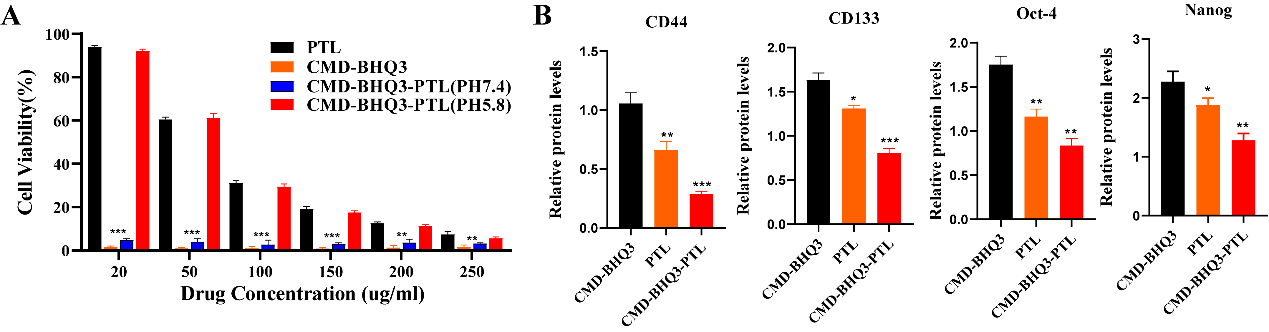


**Figure S4**. **Enhancement of stemness in CRC cells via the cellular uptake of CMD-BHQ3-PTL.** (**A**) MTT assay to investigate the impact of nanoparticles on cell viability. (**B**) WB analysis of the grayscale value histogram of stemness-related proteins. Statistical results are presented as the mean ± SD using a student’s t-test or one-way ANOVA. * P < 0.05; ** P < 0.01; *** P < 0.001.


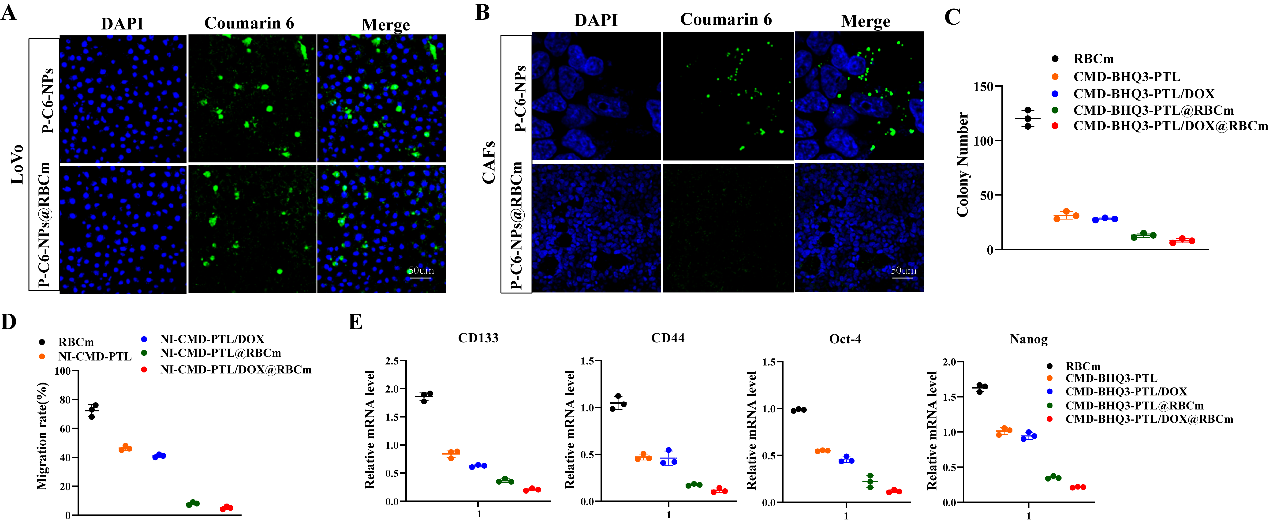


**Figure S5**. **CMD-BHQ3-PTL/DOX@RBCm inhibits CRC stem cell-induced tumor progression.** (**A**) Uptake of P-C6-NPs and P-C6-NPs@RBCM by LoVo cells. (**B**) Uptake of P-C6-NPs and P-C6-NPs@RBCM by CAFs. (**C**) Statistical analysis of colony formation assay. (**D**) Results and statistical analysis of the cell invasion assay. (**E**) RT-qPCR detection of cell stemness. Statistical results are presented as the mean ± SD using a student’s t-test or one-way ANOVA. * P < 0.05; ** P < 0.01; *** P < 0.001.


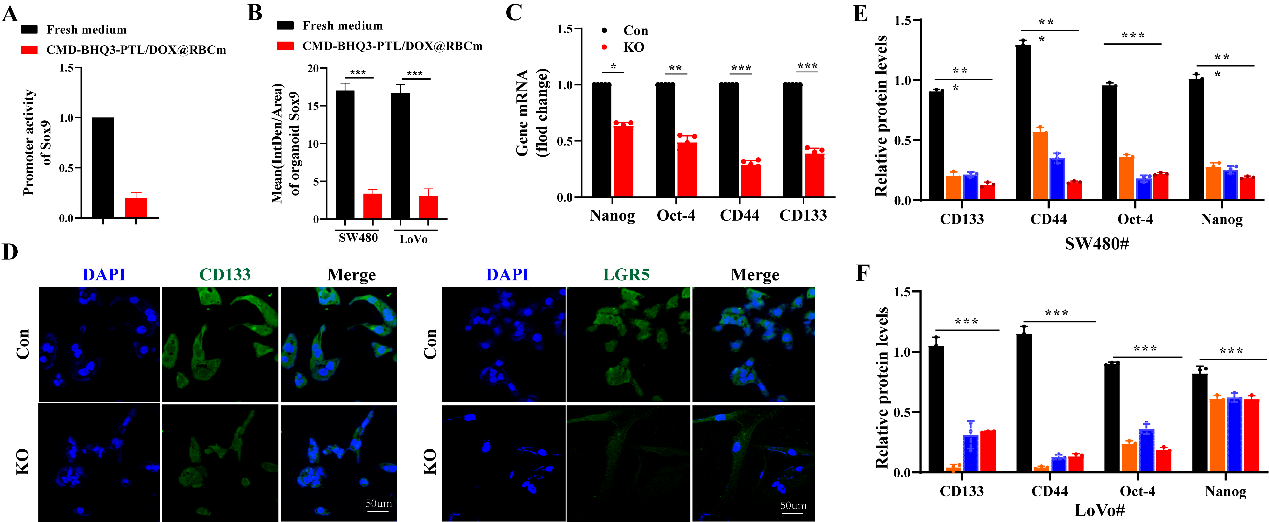


**Figure S6. CMD-BHQ3-PTL/DOX@RBCm inhibits Sox9-related CRC stemness**. (**A**) Statistical analysis of Sox9 protein expression levels in LoVo cells. (**B**) Immunofluorescence analysis of SOX9 expression. (**C**) RT-qPCR analysis of DNA expression following SOX9-KO. (**D**) Immunofluorescence results for the stemness-related genes CD133 and LGR5 in LoVo cells after SOX9-KO. (**E-F**) WB analysis to assess the impact of CMD-BHQ3-PTL/DOX@RBCM intervention on stemness-related protein expression in SOX9-KO LoVo cells. Statistical results are presented as the mean ± SD using a student’s t-test or one-way ANOVA. * P < 0.05; ** P < 0.01; *** P < 0.001.


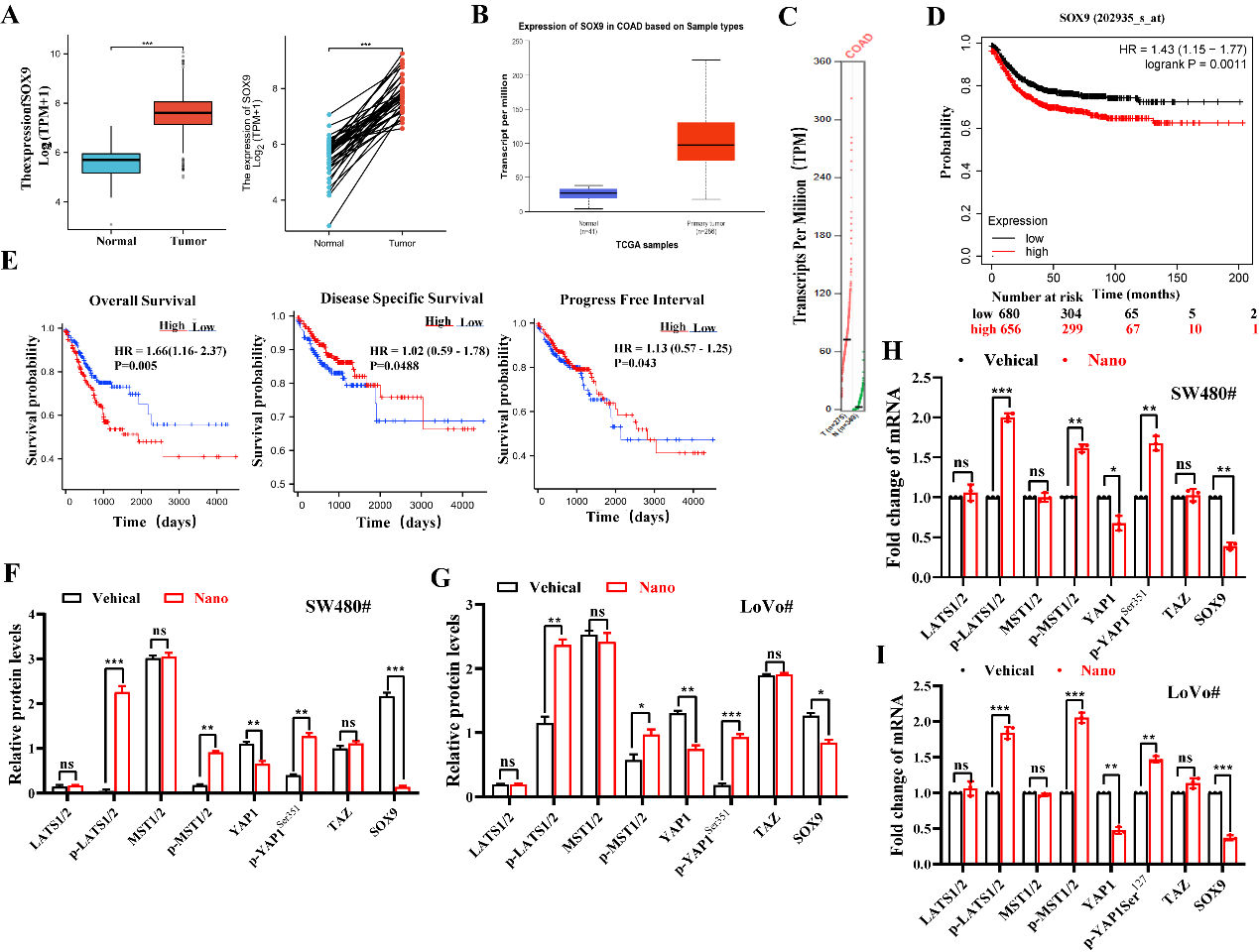


**Figure S7. CMD-BHQ3-PTL/DOX@RBCm suppresses SOX9 expression in CRC by inhibiting activation of the Hippo/YAP1 signaling pathway.** (**A**) TCGA Database, (**B**) UALCAN, and (**C**) GEPIA, along with bioinformatics analysis from the Xiantao Academic website, were used for SOX9 expression analysis in normal and tumor tissues. (**D**) GEPIA was employed to assess the correlation between SOX9 levels and the survival period of patients with gastric cancer. (**E**) The Xiantao Academic website was used to examine the correlation between SOX9 levels and the survival period of patients with gastric cancer. (**F-G**) WB analysis displaying grayscale statistical graphs of protein expression in the Hippo/YAP1 pathway after nanoparticle intervention. (**H-I**) RT-qPCR analysis presenting statistical graphs of gene expression in the Hippo/YAP1 pathway after nanoparticle intervention. Statistical results are presented as the mean ± SD using a student’s t-test or one-way ANOVA. * P < 0.05; ** P < 0.01; *** P < 0.001.


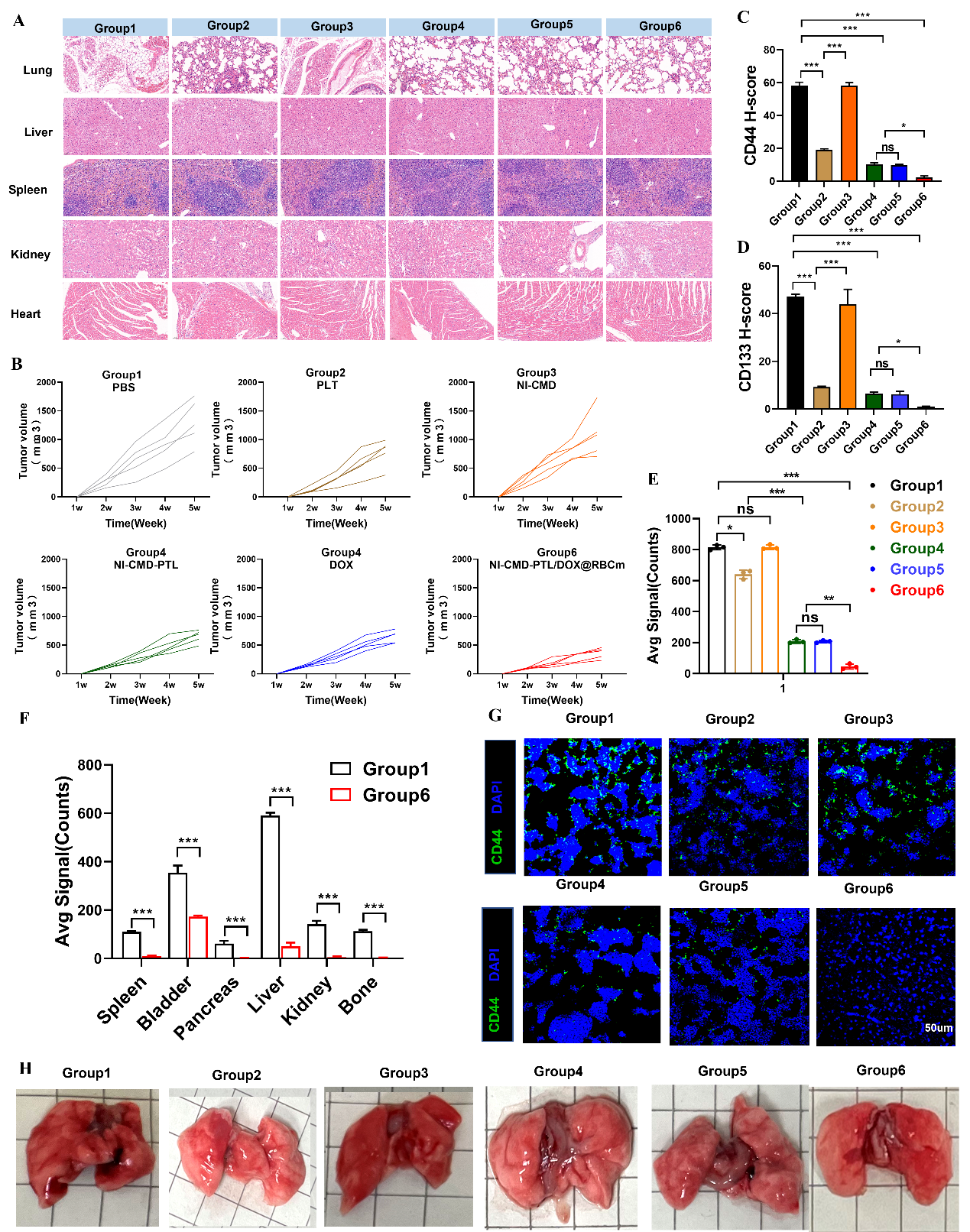


**Figure S8. CMD-BHQ3-PTL/DOX@RBCm inhibits CRC metastasis by regulating cancer stemness.** (**A**) Immunohistochemical analysis was performed to assess the biosafety of the therapy in each treatment group. (**B**) Tumor volume statistics for the mice in each group are presented in the graph. Immunohistochemical analysis and statistical charts for (**C**) CD44 and (**D**) CD133 expression in tumors from each group of mice are presented. (**E**) Semi-quantitative statistical maps of *in vivo* mouse imaging. (**F**) Semi-quantitative statistical map of fluorescence imaging of an isolated organ. (**G**) Immunofluorescence results depicting CD44 expression in tumor tissues of mice from each group. (**H**) Lung metastasis status of the mice in each experimental group. Statistical results are presented as the mean ± SD using a student’s t-test or one-way ANOVA. * P < 0.05; ** P < 0.01; *** P < 0.001.


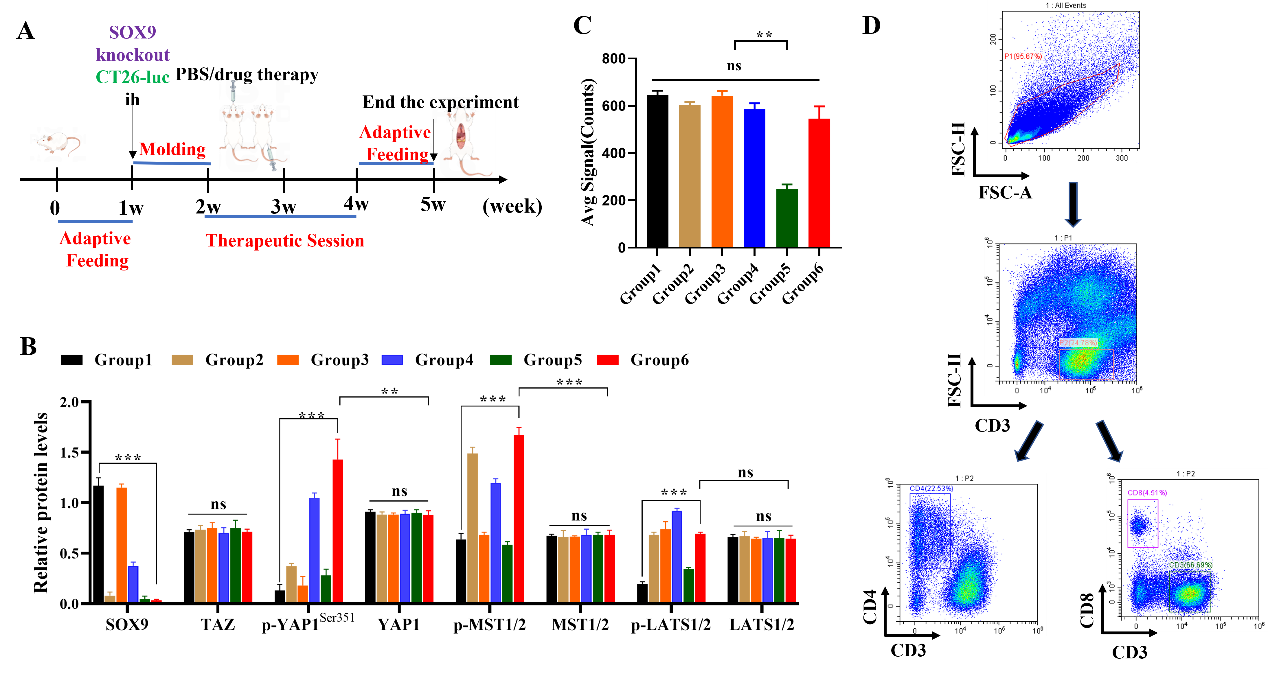


**Figure S9. CMD-BHQ3-PTL/DOX@RBCm modulates tumor stemness and suppresses tumor metastasis via the Hippo/YAP1/SOX9 pathway.** (A) Workflow for establishing the mouse metastasis model. (B) Quantitative analysis of grayscale values for core protein expression in the Hippo/YAP1/SOX9 pathway using WB. (C) Semi-quantitative analysis of the *in vivo* imaging of tumors formed in SOX9-KO mice. (D) Flow cytometry gating strategy. Statistical results are presented as the mean ± SD using a student’s t-test or one-way ANOVA. * P < 0.05; ** P < 0.01; *** P < 0.001.

**The full uncropped Gels and Blots images**

**Figure1**


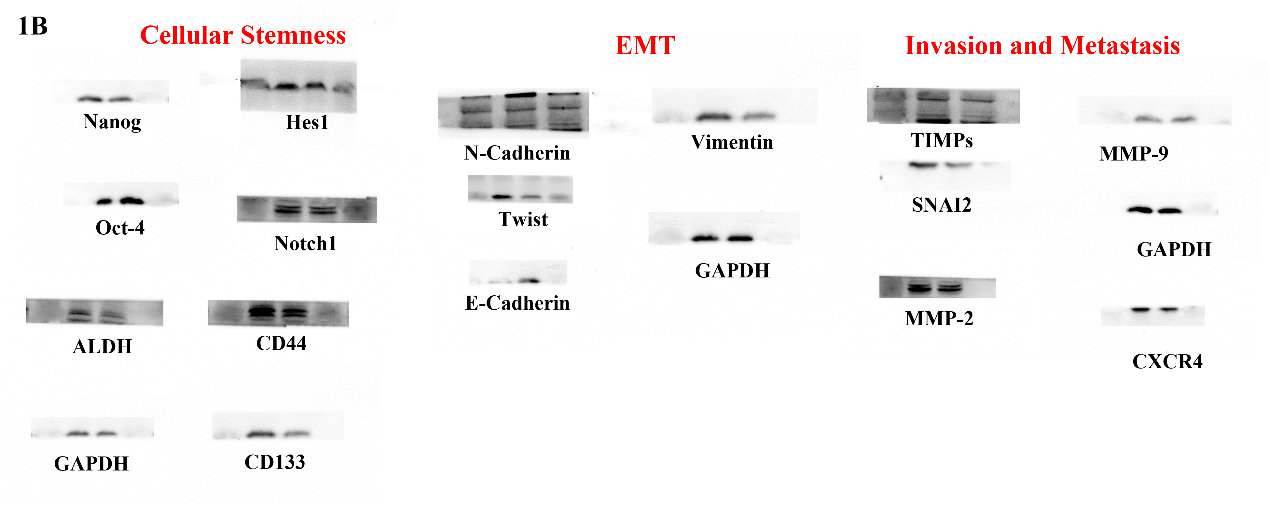


**Figure S1**


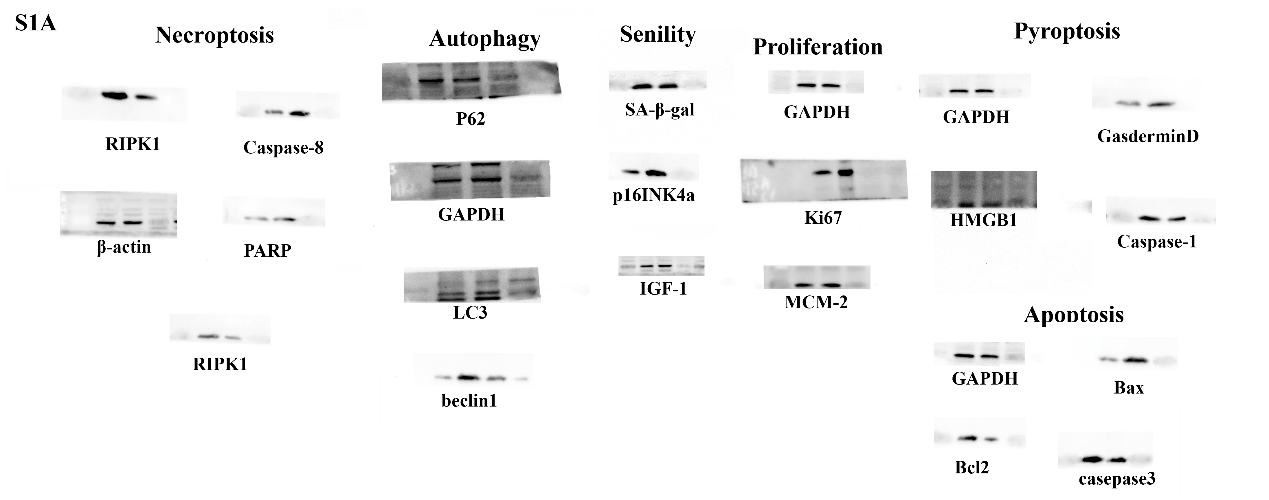


**Figure4-5**


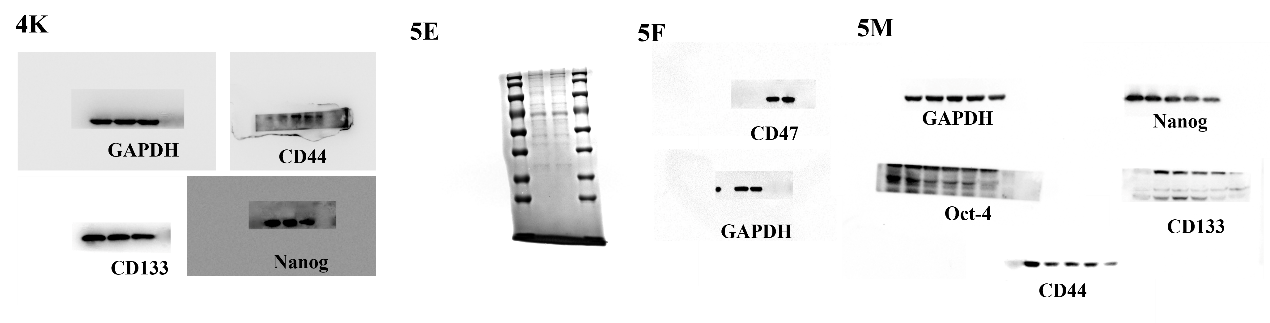


**Figure6-S6**


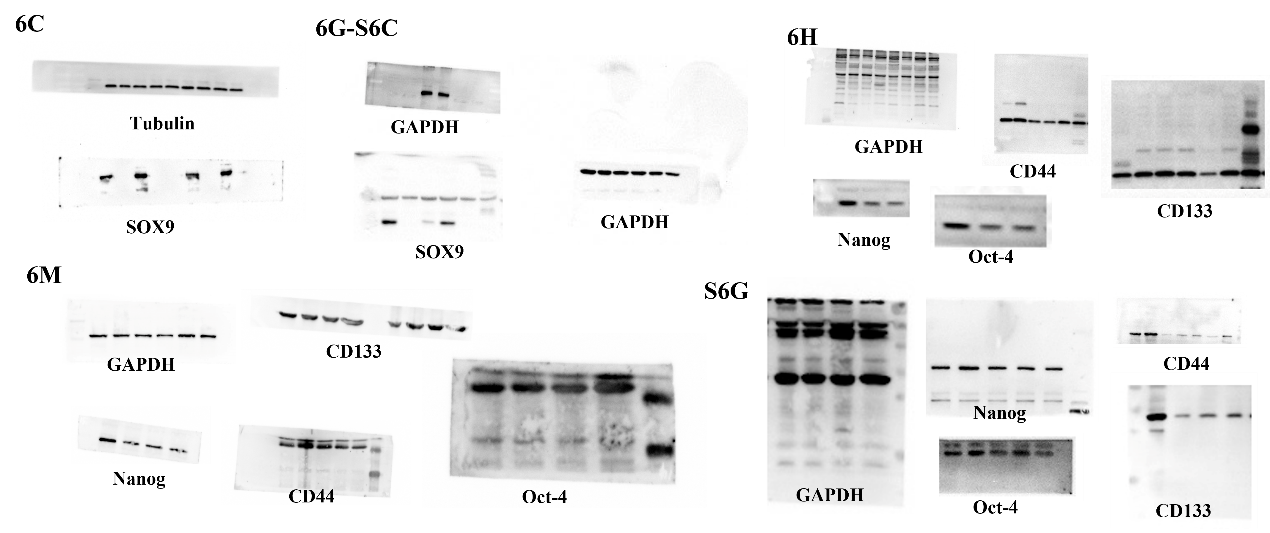


**Figure7**


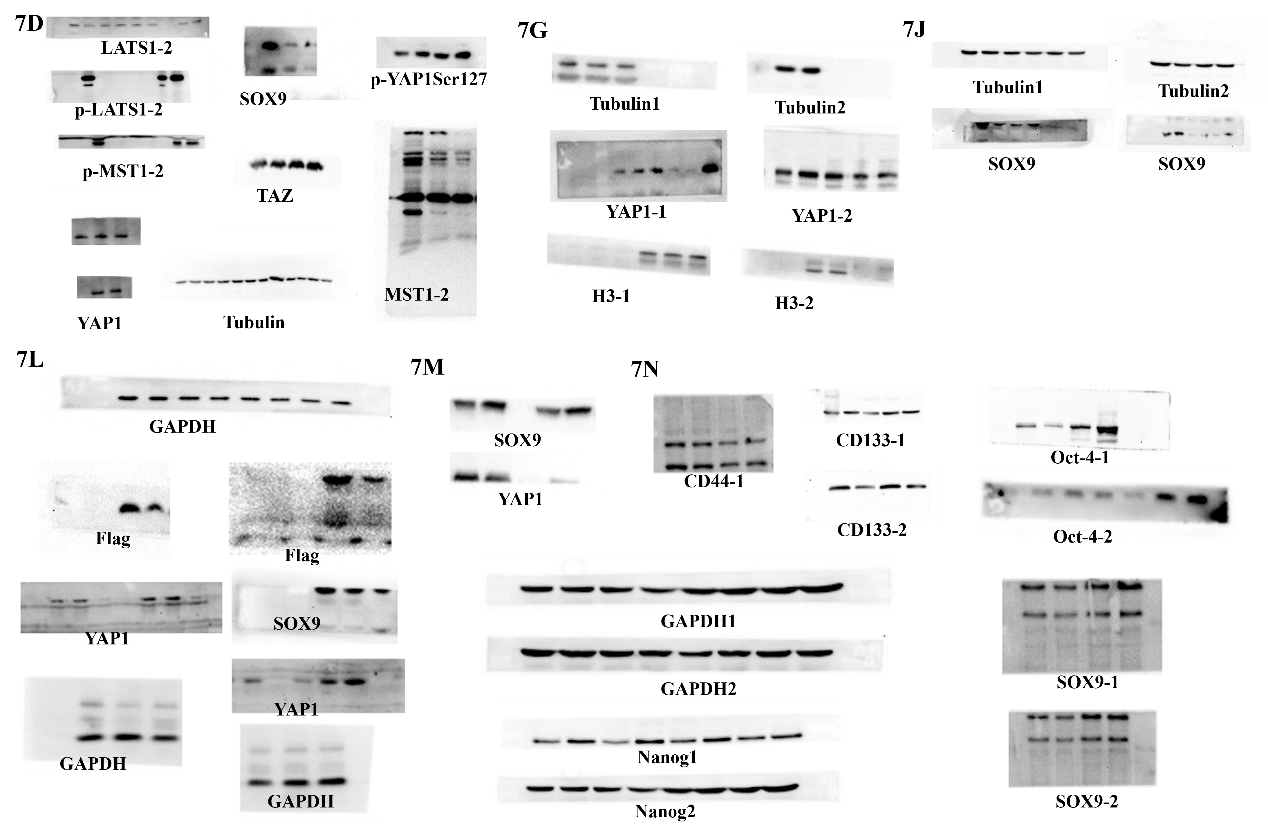


**Figure8-9**


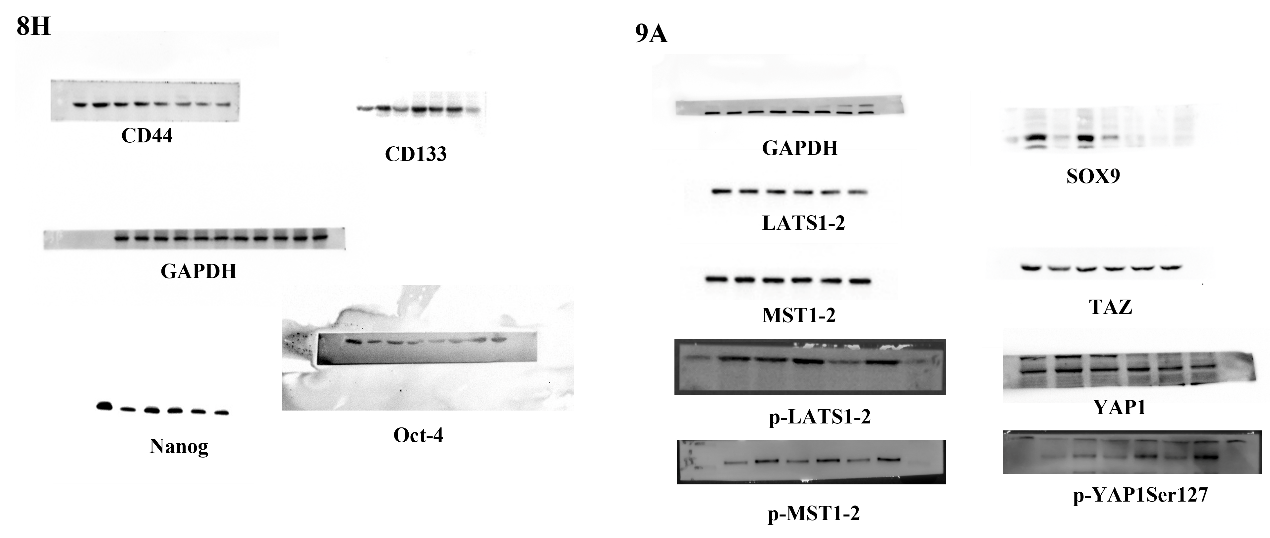

Supplement: Supplementary file 1 — Supporting Information [file SMLL-21-2407191-s001.docx]
